# Sustainable and scalable double slope solar still: a comprehensive experimental assessment of energy, exergy, economic, environmental, sensitivity and distillate performance

**DOI:** 10.1038/s41598-026-40989-3

**Published:** 2026-02-26

**Authors:** Ramasamy Dhivagar, Perumalsamy Jidhesh, Sung Chul Kim, Dhinesh Balasubramanian, Utku Kale, Artūras Kilikevičius

**Affiliations:** 1 School of Mechanical Engineering, Yeungnam University, 280 Daehak- Ro, Gyeongsan, 712-749 Gyeongbuk South Korea; 2 Department of Mechanical Engineering, Karunya Institute of Technology and Sciences, Coimbatore, 641114 India; 3 Department of CAE, Renault Nissan Technology and Business Centre India, Chengalpattu, 603002 India; 4 Department of CAE, Onward Technologies, Chennai, 600041 India; 5 Department of Port Engineering, Lithuanian Maritime Academy (LMA), Vilnius Gediminas Technical University, Klaipėda, Lithuania; 6 Department of Aeronautics and Naval Architecture, Faculty of Transportation Engineering and Vehicle Engineering, Budapest University of Technology and Economics, Műegyetem rkp. 3, Budapest, 1111 Hungary; 7 Mechanical Science Institute, Vilnius Gediminas Technical University, Plytinės g. 25, Vilnius, 10105 Lithuania

**Keywords:** Double-slope solar still, Electrical heating element, Enviro-economic assessment, Phase change material, Productivity, Solar photovoltaic panel, Thermodynamic analysis, Energy science and technology, Engineering, Environmental sciences

## Abstract

In double-slope solar stills (DSS), a significant amount of solar energy is wasted as heat and reducing the system freshwater generation efficiency. To overcome this, a reconstructed double-slope solar still (RDSS) with a photovoltaic panel (PV) mounted on top is proposed which facilitates for simultaneous generation of freshwater, heat, and electricity. The PV panel partial shading is reduced by transferring the generated power to a heating element that preheats the inlet saline water, while paraffin wax phase change material (PCM) improves thermal storage. The experimental results indicated that the RDSS substantially enhanced productivity by 92.2%, with energy and exergy efficiencies improvement of 44.2% and 46.9%, respectively. The PV panel peak power output was 47.1 W, and the system had an efficiency of 14.1% (electrical), 37.1% (thermal), 88.5% (heating element), 52.1% (overall energy), and 5.9% (overall exergy). Economically, the RDSS reduced the cost per litre (CPL) of distillate by 17.3% and the payback period (PBP) by 18.2% than DSS which indicating higher financial feasibility. Additionally, the environmental study revealed that the RDSS achieved 98.2% more CO₂ reduction than DSS. In sensitivity analysis, the extending system lifespan from 5 to 25 years lowered production costs by 73.1% at a 2.5% interest rate, but CO₂ mitigation and carbon credit earned (CCE) elevated by 536%. Moreover, the RDSS had a 1.6% higher sustainability rating than DSS, though both systems generated potable water that met world health organization (WHO) requirements. Finally, these findings support RDSS as a low-cost, ecologically friendly and scalable alternative for decentralized freshwater and energy generation.

**Supplementary Information:**

The online version contains supplementary material available at 10.1038/s41598-026-40989-3.

## Introduction

The demand for innovative and sustainable techniques to provide a consistent supply of freshwater has grown as a result of exploitation of water resources, increasing groundwater depletion, and rising pollution levels. Desalination is required to produce drinking water because the majority of the world’s water is salty. Traditional technologies such as reverse osmosis, multistage flashing, and multi-effect distillation are effective, but they require a lot of energy and use fossil fuels, which contribute to global warming^1^. Renewable desalination is now the focus of research, with solar energy emerging as the most viable, cheap, and environmentally friendly approach. Solar stills are a simple and environmentally beneficial way to convert saline water into freshwater through evaporation and condensation. Even though they are inexpensive and simple to use, solar stills have low thermal efficiency and operate intermittently because they only require daylight^2^. Some solutions to these challenges include adding additional solar collectors, modifying the still design, and using thermal energy storage systems such as PCMs to store heat throughout the day and release it at night to sustain freshwater output.

Modern research focuses on improving DSS production via heat management and design changes. Saha et al.^3^ improved DSS performance using vacuum and paraffin wax PCM. The system achieved a maximum production of 7 L/m^2^/day, which is 22.3–63% greater than standard DSS. According to this study, the combination of PCM with vacuum could increase energy efficiency by 28.7%. Anburaj et al.^4^ evaluated DSS performance with various wick materials and energy storage technologies. The highest distillate production of 3.1 L/m^2^/day was achieved with sponge and copper materials, which was 17.5% and 12.4% higher than DSS setups with jute-copper and cotton-copper. The sponge-copper DSS achieved the highest average energy efficiency of 51% with outperforming the cotton-copper and jute-copper combinations by 18.6% and 10.9%, respectively. Murad et al.^5^ employed five spinning cylinders and four PV-powered heaters to enhance evaporation surface area while decreasing water depth, hence improving DSS performance. Using PV-powered heating increased productivity by 6 L/m²/day (221.3%) compared to 3.2 L/m^2^/day (95%) without it. The findings suggested that such design adjustments can increase DSS performance in a long-term and cost-effective manner with making them ideal for remote deployment. Thavamani et al.^6^ found that trapezoidal channels in DSS have increased thermal efficiency. The modified configuration improved heat absorption and evaporation dynamics which boosted production by 30.4%. Dhaoui et al.^7^ discovered that cylindrical fins on the absorber plate significantly increased DSS output. The maximum fin diameter (80 mm) increased it by 14.1% than simple design with 3.3 L/m^2^/day. These findings demonstrated that the way fin-assisted setups increase evaporation and total freshwater production.

Furthermore, Ghazy^8^ experimented a DSS with a solar air heater condenser to recover thermal losses and create productivity and heated air. The simulations indicate that the proposed system outperformed conventional stills in thermal efficiency by 15% in forced circulation and 6% in natural circulation, with minimal productivity variations. Ahmed et al.^9^ examined productivity and cost-efficiency trade-offs in solar still desalination. The PCM and nanofluid integration increased the productivity by 87.4% and 350%, respectively. It reviewed the decision-support tool for water cost and performance-based system selection and highlights unique designs including PV panels, fins, and wick materials. The multidisciplinary approaches were suggested to boost solar desalination efficiency and sustainability. For off-grid greenhouses, Shelake^10^ examined solar-driven desalination methods. The study reviewed thermal efficiency and productivity of single-slope, double-slope, pyramid, and stepped solar stills. The performance beyond peak sunlight was enhanced by integration with PV systems and PCMs. This review also highlights solar stills as sustainable, low-cost freshwater sources for decentralized delivery in arid and semi-arid locations. Widatalla et al.^11^ studied a low-cost solar still with a transparent PV panel for freshwater and energy generation. The performance was optimized using simulations software. At 3 m/s wind velocity, increasing saline water temperature from 60 to 70 °C boosted productivity by 67% and to 90 °C by 141%. The method also could generate water and electricity sustainably in isolated areas. Dubey and Arora^12^ found that incorporating materials like paraffin wax and Al₂O₃ nanoparticles enhances thermal storage and heat transfer in DSS. It was increased productivity by 60.4% and energy efficiency by 68.3% over traditional stills. The review also showed that hybrid PCM composites improved the latent heat utilization and DSS performance through facilitating sustainable solar desalination.

Several research have examined that hybrid heating systems improve solar still performance. Dawood et al.^13^ found that integrating a solar still with a 450 W electrical heater significantly increased productivity. The heater raised it to 12.8 L/m^2^/day in experiments. It also increased basin temperatures under low solar irradiation which accelerating evaporation and enhancing efficiency. Feria-Diaz^14^ studied a moderate hybrid DSS with a 300–500 W electrical heater which resulting in a 6.8 L/m²/day gain in daily productivity. Despite low solar irradiation, the regulated heater-maintained basin temperatures for continuous evaporation. This hybrid technique multiplied water output and improved energy use by proving its reliable desalination potential. According to Dhivagar et al.^15^, a trigeneration DSS with a PV-powered electrical heater increased daily productivity from 2.5 to 3.4 L/m^2^/day. The study demonstrated that the practical benefits of PV-powered heating and solar stills and proposes that phase transition materials and IoT-based optimization can improve sustainable desalination. Abdel-Aziz et al.^16^ improved the performance of solar still by utilizing paraffin wax PCM and a PV-powered electric heater which resulting in 3.1 times more productivity in spring and 2.67 times more in summer at 65 °C. It also increased PCM heat transmission, productivity, economic performance and water savings. The DSS experiment with micro-encapsulated PCM by Afolabi et al.^17^ generated 7.5 L/m²/day of productivity which had 105% increase over the conventional still. The PCM allowed for 3 h of operation after nightfall which resulting in improved evaporation with basin and humid air temperatures reaching 79.5 °C and 77.5 °C, respectively. It also minimized heat losses and maintained WHO-compliant water quality by making it practical and sustainable.

Moreover, Aftiss et al.^18^ compared three solar still systems with PCM and storage tanks quantitatively. The advanced system (still-III) achieved 8.6 L/m^2^/day productivity in Marrakech and up to 76.1% energy efficiency due to latent heat release extending evaporation after nightfall. The winter harvests were decreased due to ambient temperature constraints. In dry climates, the PCM and storage tanks were excellent solar desalination methods, according to the study. Almutlaq^19^ investigated a 3D-printed horizontal diffusion solar still with 400–1000 W/m^2^ electric heating. The five-effect arrangement has a maximum productivity of 1.9 L/m^2^/day and a solar-to-vapor efficiency of 107% by improving with more stages. According to annual forecasts, Dhahran output peaks at 10.2 L/m^2^/day in June. The controlled electric heating models and improves solar still performance was revealing multi-effect system optimization opportunities. Elbar et al.^20^ compared a PV panel solar still with conventional solar still through 4E analysis. The solar module on the still backside boosted basin temperature by 23% and daily productivity by 52.3%. The PV integration also increases thermal behavior, while preserving compact land usage and sustainable operation. Lee et al.^21^ created a multi-effect diffusion solar distiller with horizontal grooved wick-free plates. It demonstrated 7% improved productivity and stability despite changing feed flow and achieving 84% energy efficiency at 10.8 MJ/m² solar irradiation using a three-effect setup. The design decreases thermal resistance, improves performance, and boosts productivity. Conserva et al.^22^ mounted wind-powered thermoelectric heating modules in solar still basins for 24 h operation. In the hybrid system, productivity increased to 7.6 L/m²/day which demonstrated sustainable desalination.

Advanced thermodynamic analysis is needed to optimize hybrid solar desalination systems since it evaluates energy and exergy performance which influences efficiency and sustainability. Sun et al.^23^ found that geometric adjustments improve heat gradients and vapor formation in a modified DSS to 6.3% greater exergy efficiency compared with simple DSS. Sethi et al.^24^ tested the forced circulation in DSS and found 0.3% to 1.3% exergy efficiency in 24 h experimentation which demonstrating the importance of water circulation and flow management on thermal performance. Saragi and Damanik^25^ examined a DSS under different basin design, water production, ambient temperature with solar irradiation and found exergy efficiencies from 0.9% to 5.4% by showing that environmental and operating variables affect it. Sharshir et al.^26^ integrated carbon black nanoparticles and linen wicks to maximize exergy efficiency in a stepwise DSS by raising water temperatures and increasing evaporation fractional exergy. In this, the nanomaterials and wick enhancements improved the heat transfer and thermal storage. Vigneswaran et al.^27^ added an acrylic auxiliary condensing surface beneath the top glass cover which raised exergy efficiency from 1.6% to 2.1% compared to a typical solar still. Rastegar et al.^28^ found that hybrid thermal systems using water heater with thermosyphon heat exchanger increased exergy efficiency by 41% by recovering waste heat and prolonging operational hours. Bait^29^ studied the quantitative analysis of a DSS using tubular heater and demonstrated 41% global exergy efficiency which focusing hybrid system energy efficiency and sustainability. Naveenkumar et al.^30^ used the vacuum fan (solar powered), external condenser, and nanofluids in a DSS to increase exergy efficiency by 78.6% by showing the synergy of heat transfer and active ventilation. Radhakrishnan et al.^31^ observed DSS exergy efficiencies of 2% to 3.5% in Oman which demonstrating that local weather affects performance. Finally, Hassan^32^ showed that DSS had 3.3% greater exergy efficiency than simple solar stills in summer by proving that multi-slope designs perform better in high solar irradiation. These studies showed that exergy-based thermodynamic analysis is essential for improving design, incorporating PCMs, improving condensation and evaporation, and integrating hybrid thermal systems to boost solar desalination technology productivity, efficiency and sustainability.

The productivity cost is affected by primary resource expenditure, setup effectiveness, system operation and maintenance costs in economic analysis. Rahbar et al.^33^ showed that DSS with PV-powered thermoelectric modules boosted productivity by 3.2 times while being economically viable. Over 12 h, the system used 1 kWh and produced CPL at 0.142 $ during daylight and 0.237 $ at night which resulting in a cost-effective trade-off for increased productivity. Shoeibi et al.^34^ tested a DSS with a thermoelectric system to cool the glass cover and heat the basin. The improved system increased productivity and reduced CPL from 0.176 $ to 0.105 $ for the passive still. The study showed that thermoelectric integration makes solar desalination economically viable despite its complexity and energy requirements. Almajali et al.^35^ built a DSS with evacuated tubes, external reflectors, and a pump (PV powered) that performed well at 0.005 $. Similarly, Tuly et al.^36^ used fins and reflectors with nano-PCM in a DSS and observed 95% lower CPL and 92% lower PBP. Samuel et al.^37^ tested wicks in hybrid water heater solar still and found that solar PV panel had strong productivity boost by 245% than simple DSS with low maintenance costs of 117–283%. Shajahan et al.^38^ analyzed the cost effectiveness in PCM integrated DSS was 16% lesser than simple DSS with optimum productivity and thermal effectiveness. Zayed et al.^39^ used spraying nozzle in a DSS and observed the CPL of 0.013 $ which far lower than DSS. This study found that the effective lower PBP observed in most of the solar energy powered systems with minimized heat usage.

Technical sustainability is assessed by combining economic viability and environmental effect which mainly focusing on CO₂ emissions reductions. The study by Yugbodh et al.^40^ found that using stainless steel curl strips in an altered desalination system had 12% of reduced CO₂ emissions than aluminum fins with showing the impact of material choices on environmental performance. Singh^41^ used the PV panel in flat-plate collectors in a DSS which increasing freshwater productivity by 25.1%, exergo-economic performance by 54.6%, and enviro-economic efficiency by 92.6%. The annual CCE of modified DSS using black cement fins covered by cotton was 54.1% higher than simple DSS, according to Vishwanath et al.^42^ In this, the thermal absorber modification was maximized the energy use. In a study by Arunkumar et al.^43^, pyramid solar stills reduced 15% of lifetime CO₂ emissions than conventional type, with an annual CCE of 0.5 and substantially higher than the normal 0.2 of traditional designs. According to Tripathi et al.^44^, setups using PCMs reduced CO_2_ emissions by 8.3 tons per operational cycle compared to conventional approaches. The environmentally optimized designs of DSSs with PCMs demonstrate their promise for low-carbon freshwater generation. Additionally, Abhishek and Rajesh^45^ used the evacuated tubular collectors in conical solar still and found the reduced environmental economic costs by 81.1% which indicating improved sustainability and cost-effectiveness.

Recent studies have proposed advanced solar still designs to overcome the low productivity of CSS by enhancing solar input and condensation. Elminshawy et al.^46^ developed a modified floating solar still integrated with a Fresnel lens and an external condenser, achieving up to 18.9 L/m^2^/day, which corresponds to a 470% increase in productivity, along with markedly improved energy and exergy efficiencies. Bady et al.^47^ proposed an enhanced conical solar still incorporating phosphate-filled copper fins as a porous sensible heat storage medium to improve evaporation. The optimized configuration achieved a maximum yield of 8.2 L/m²/day, representing a 70% increase over the CSS, with thermal efficiency rising from 54.7% to 84.6% and annual CO₂ mitigation increasing from 2.1 to 3.5 tons. Similarly, Soliman et al.^48^ enhanced the performance of CSS by integrating stainless-steel balls as sensible heat storage and coupling it with a parabolic trough concentrator. The optimized system achieved 5.6 L/m²/day, representing a 76.8% productivity increase over the CSS, along with notable improvements in CO_2_ mitigation and CPL, indicating improved thermal and enviro-economic performance. El-Ghandour et al.^49^ investigated a modified solar still incorporating a separate external storage system with a Fresnel lens and solar thermal collector to enable daytime energy storage and nighttime operation. The optimized configuration achieved 4.1 L/m^2^/day with an average energy efficiency of 25.9%, while maintaining low CPL ($0.0119/L) and a short PBP, showing the effectiveness of external thermal storage. These studies indicate that strategic design changes, material selection, renewable energy integration, and thermal energy storage can improve solar desalination systems’ environmental and economic performance.

## Objective and novelty of this study

This study offers a new experimental setup of an RDSS combined with a double slope solar still, solar PV panel and paraffin wax PCM which is a creative method that hasn’t been documented in the general scientific literature before. Traditional active solar stills require several external components including PV panels, solar collectors, and pumps, which increases system complexity, maintenance, and startup costs. Indirect heat transfer also contributes to energy losses in these systems^50^. The proposed design places the solar PV panel right above one DSS side, enabling continuous power generation and passive heat transfer in a compact unit. In order to increase productivity without using outside energy sources, the electricity produced by PV panel is used immediately to preheat the incoming saline water through an electrical heating device. This preheating method counteracts the shadowing effect that limits solar irradiation on the still surface and productivity.

**Fig. 1:**
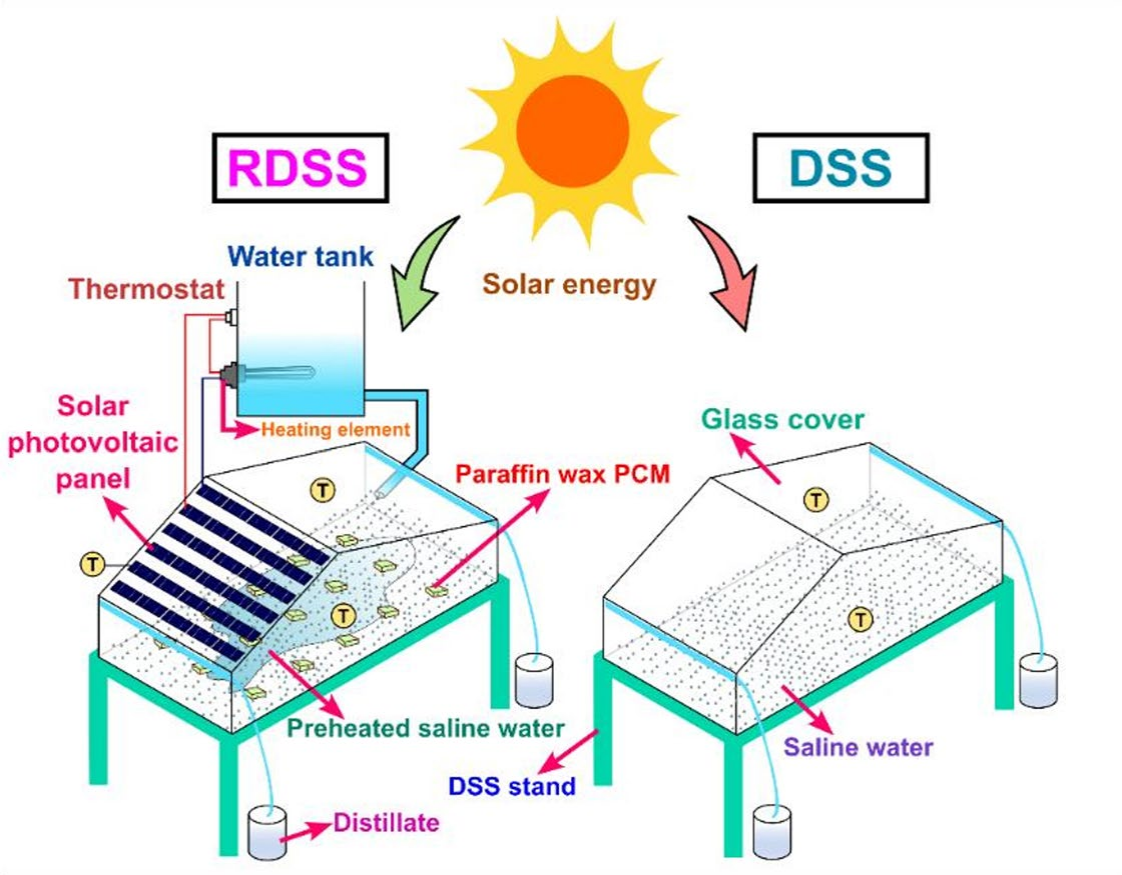
Schematic view of RDSS with DSS in this proposed study.

Furthermore, the use of paraffin wax PCM improves thermal energy storage by absorbing extra heat during the strongest solar hours and releasing it gradually during less intense. This prolongs the productivity period, maintains the basin temperature, and enhances the system overall functionality. The proposed system integrates PCM-based thermal storage, PV-powered heating element preheating, and direct coupling of solar PV panel to overcome traditional designs’ large spatial requirements, energy losses from subsystems, and efficiency declines due to shadowing. It offers an environmentally beneficial, low-maintenance, and sustainable off-grid alternative by producing distillate, storing thermal energy and producing electricity all at once. To the best of the authors’ knowledge, this is the first experimental study to integrate DSS with preheated saline water, PCM, and direct solar PV panel coupling in a single system to enhance energy efficiency, exergy performance, and economic and enviro-economic viability with distillate analysis while minimizing shadowing effects. Figure 1 depicts the schematic view of RDSS in this proposed study.

## Experimental work

**Fig. 2:**
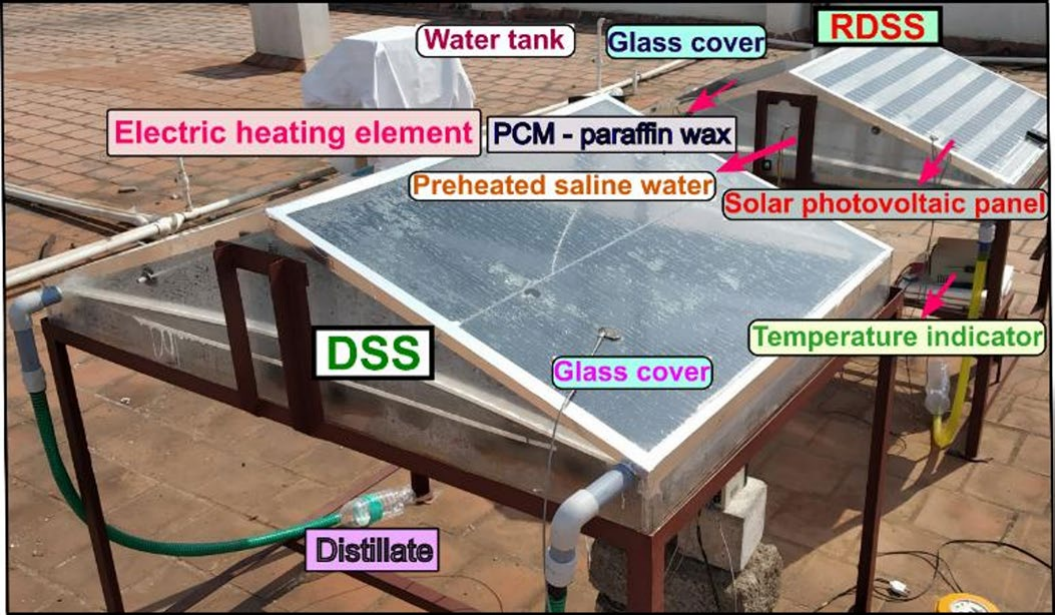
Experimental view of RDSS and DSS.

During the experiment, two DSS with similar dimensions were built to assess performance. The trials were conducted on May 30, 2024, at Coimbatore, India (11.0168° N, 76.9558° E). The first setup served as a standard reference unit, whereas the second was a RDSS with PV panel mounted on another top portion of the system. In RDSS, the PV panel electrical output was used to power a direct current heating element, which was immersed in a 15 L saline water tank to preheat the feedwater before it entered the basin. In this tank, a fixed volume of preheated feedwater was delivered at predetermined intervals while keeping a constant basin depth to guarantee repeatability and equitable comparison across all the experiment tests. This direct contact solution enabled the effective transfer of thermal energy from PV-generated power to stored saline water. To ensure passive operation, the experimentation was not utilized any mechanical pump to transport inlet preheated saline water into RDSS. rather, this was done manually. This design decision was intended to prevent external energy inputs as well as the 50 W PV module constrained power capability which was solely dedicated to the heating element. Adding a mechanical pump would have increased power consumption which decreased the heating efficiency and limited the system usefulness for small-scale applications. Maintaining a basic setup with better reproducibility and ensuring adherence to the study objectives. Figure 2 shows the experimental setup for both RDSS and DSS.

For both stills, the basin was made from 2 mm thick acrylic sheets with a surface area of 1 m^2^. A 4 mm thick glass cover was fixed at a 15° inclination with regard to the horizontal which matching the site latitude to maximize solar irradiation capture^51^. Each DSS unit was sealed with a rubber seal to prevent vapor leakage at the joints. Both systems were kept immobile and facing south to maximize solar irradiation^52^. Moreover, in RDSS, the quantity of paraffin wax PCM usage was kept at 0.5 kg and the properties are followed by: melting point 58–60 °C, latent heat of fusion 190 kJ/kg, thermal conductivity 0.24 W/m-K, and specific heat capacity 2.1 kJ/kg-K^53^. Additionally, the monitored saline water thickness was 2 cm in the both systems and condensed distillate trickled down the inside sides of the glass cover via small channels before collecting in a storage jar. In RDSS, a solar PV panel with a surface area of 0.5 m² and a nominal power capacity of 50 W was kept above the glass cover of the still to capture solar energy while producing electricity. Under peak solar irradiation, the PV panel generated a maximum power output of 50 W, which corresponded to an operating voltage of approximately 17.3 V and a current of 2.9 A. The panel open-circuit voltage and short-circuit current were measured to be at 21.4 V and 3.1 A, respectively which had a fill factor of 0.72 and an overall conversion efficiency of 14.1%.

**Fig. 3:**
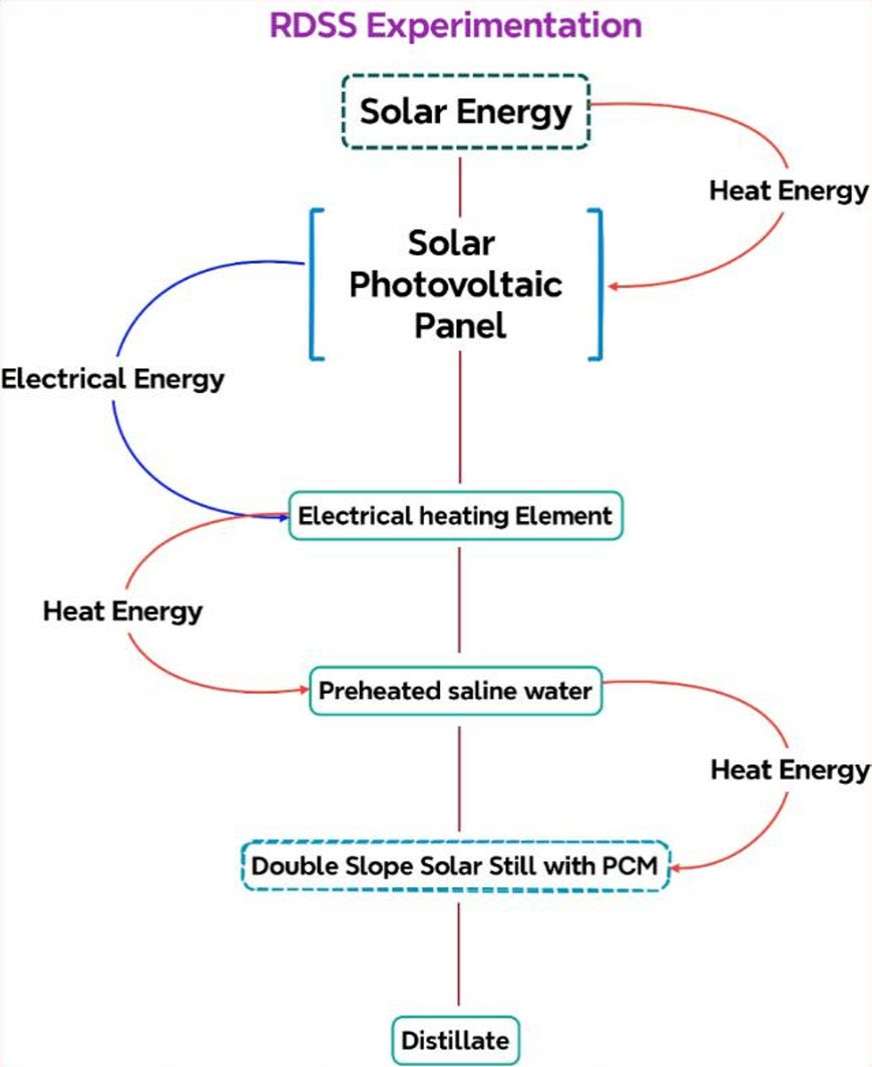
Heat and electrical pathways of RDSS experimentation.

The shadowing impact that the PV panel physical presence created on the solar still surface, however, tended to decrease thermal absorption and consequently freshwater productivity. In order to get over this limitation, the PV panel electrical energy was used to warm the saline water at the entry point. A 12 V direct current nichrome resistive heating element inserted in a silicone rubber pad (40 mm diameter, 200 mm length, immersion type) was connected to the PV panel to accomplish this. The heating element was made to work in accordance with the output characteristics of the PV panel with a rated power of 45–50 W, an electrical resistance of roughly 2.8–3.2 Ω, and an operating current of 3.3 A. The unit could endure temperatures between 100 and 200 °C with a thermostat used for safe and dependable operation. In order to make up for productivity losses brought on by the shadowing impact and enhance the overall performance of the RDSS, the PV–heating element integration made sure that the solar panel electrical output was effectively used for direct thermal enhancement of the saline feedwater. Figure 3 illustrates the heat and electrical pathways of RDSS experimentation.

The experimental data were collected on an hourly basis from 09:00 to 18:00 h throughout the testing period. The temperatures of the saline water, glass cover, air–vapor mixture, paraffin wax PCM, preheated water, ambient air, and both surfaces of the solar PV panel were monitored using type-K thermocouples with an accuracy of ± 0.1 °C. The incident solar irradiation was measured by a pyranometer (precision ± 10 W/m^2^), while the wind velocity was recorded with an anemometer (accuracy ± 0.2 m/s). To evaluate the electrical output of the PV module, a digital ammeter (± 0.001 mA) and a voltmeter (± 0.1 V) were employed. The distillate yield was quantified hourly using a calibrated measuring flask (± 5 ml). The overall uncertainty of measurements was calculated according to the methodology reported as follows^53^:

1 $$u=\frac{a}{{\sqrt 3 }}$$

where u represents the standard uncertainty and a denotes the instrument accuracy. The measurement uncertainties of all instruments employed in the study are summarized in Table 1 and the combined uncertainty of the derived parameters were determined using the following relation^54^:

2 $$u\left( y \right)={\left[ {{{\left( {\frac{{\partial y}}{{{x_1}}}} \right)}^2}.{u^2}\left( {{x_1}} \right)+{{\left( {\frac{{\partial y}}{{{x_2}}}} \right)}^2}.{u^2}\left( {{x_2}} \right)+ \ldots } \right]^{0.5}}$$

where y presents a function of the input amount of the numbers xi and u(xi). Based on this, the average uncertainties in productivity, energy and exergy efficiency are estimated by 1.6%, 1.2%, and 0.2%, respectively.

**Table 1:** Standard uncertainty of the instruments.

Device with unit	Measuring range	Standard uncertainty
Pyranometer (W/m^2^)	0-2000	5.774
K-type Thermocouple (^o^C)	0-700	0.058
Anemometer (m/s)	0–25	0.115
Voltmeter (V)	0–50	0.058
Ammeter (A)	0–5	0.0005
Measuring flask (ml)	0-1000	2.887

## Mathematical analysis

The RDSS and DSS are mathematically assessed for thermal performance, cost-effectiveness, and environmental impact. These evaluations assess system sustainability and practicality.

### Energy efficiency

The energy efficiency measures the percentage of incident solar energy converted into freshwater by latent heat of vaporization. To calculate RDSS and DSS efficiency, evaporation rate was interrelated with net solar input. The latent heat of vaporization (*L*) is expressed as^55^:

3 $$L=2.4935~ \times {10^{6~}}~ \times ~\left[ {1 - 9.4779 \times {{10}^{ - 4}}~{T_w}+1.3132~ \times {{10}^{ - 7}} \times ~{T_w}^{{2~}} - ~4.794~ \times {{10}^{ - 9}} \times ~{T_w}^{{3~}}} \right]$$

Here, *T*_*w*_ denotes the saline water temperature. The energy efficiency of DSS (${\eta _{dss}})$ is determined using^55^:

4 $${\eta _{dss}}=\frac{{{m_w} \times L}}{{{A_{dss}} \times {I_{si}} \times 3600}}$$

where, *m*_*w*_ represents the cumulative distillate in litre, *A*_*dss*_ is the effective area of the solar still, and *I*_*si*_ denotes the solar irradiation intensity.

For RDSS, the solar PV panel efficiency (${\eta _{pv}})$ is calculated by^51^:

5 $${\eta _{pv}}=\frac{{{P_{max}}}}{{{A_{pv}} \times {I_{si}}}}$$

where, the maximum electrical power $({P_{{\mathrm{max}}}}$) in W is estimated by multiplying the open circuit voltage (V_oc_) and short circuit current (I_sc_) by the 0.72 fill factor at the specific instance^51^. Furthermore, ${A_{pv}}$ denote the solar PV panel area (m^2^).

The thermal efficiency of solar PV panel (${\eta _{tpv}})~$in RDSS is defined by^51^:

6 $${\eta _{tpv}}=\frac{{{\eta _{pv}}}}{{0.38}}$$

The heating element efficiency (${\eta _{he}})$ in RDSS is expressed as^15^:

7 $${\eta _{he}}=\frac{{{m_{sw}} \times {C_p}\left( {{T_o} - {T_i}} \right)}}{{{P_{max}} \times 3600}}$$

here, m_sw_ denotes inlet saline water in litre, C_p_ is specific heat capacity of water in kJ/kg °C, T_o_ and T_i_ is the outlet (preheated) and inlet temperature of saline water in °C.

Finally, the overall energy efficiency (${\eta _{o - ee}})$ in RDSS is given by^15^:

8 $${\eta _{o - ee}}=\left( {\frac{{{\eta _{dss}}+{\eta _{tpv}}+~{\eta _{he}}}}{3}} \right)$$

### Exergy efficiency

The exergy is the maximum beneficial work when the system is in equilibrium. The overall RDSS and DSS exergy efficiency ($\eta _{{{\mathrm{o}} - {\mathrm{ex}}}}$) is proposed by^55^:

9 $$\eta _{{o - ex}} = \frac{{\dot{E}_{{x,out}} }}{{\dot{E}_{{x,in}} }}$$

The exergy input ($\dot{E}_{{{\mathrm{x}},{\mathrm{in}}}} )~$and the exergy output ($\dot{E}_{{{\mathrm{x}},{\mathrm{out}}}} )$ is determined by^55^:

10 $$\dot{E}_{{{\mathrm{x}},{\mathrm{in}}}} = ({\mathrm{A}}_{{{\mathrm{dss}}}} {\mathrm{I}}_{{si}} + A_{{pv}} I_{{si}} )\left[ {1 - \frac{4}{3}\left( {\frac{{{\mathrm{T}}_{{\mathrm{a}}} }}{{{\mathrm{T}}_{{\mathrm{s}}} }}} \right) + \frac{1}{3}\left( {\frac{{{\mathrm{T}}_{{\mathrm{a}}} }}{{{\mathrm{T}}_{{\mathrm{s}}} }}} \right)^{4} } \right]$$

11 $$\dot{E}_{{{\mathrm{x}},{\mathrm{out}}}} = \frac{{\dot{m}_{w} \times L}}{{3600}}\left[ {1 - \left( {\frac{{{\mathrm{T}}_{{\mathrm{a}}} }}{{{\mathrm{T}}_{{\mathrm{w}}} }}} \right)} \right] + P_{{max}} {\mathrm{~}} + ~m_{{sw}} \times c_{p} \left( {T_{o} - T_{i} } \right) \times \left( {1 - \frac{{T_{a} }}{{T_{o} }}} \right)$$

T_a_ and T_s_ denotes the atmosphere and sun (6000 °C) temperature.

### Economic analysis

The financial viability is determined by capital recovery factor (CRF) and capital cost (CC) which yields fixed annual cost (FAC).

12 $$FAC=CRF \times CC$$

13 $$CRF=\frac{{i{{\left( {1+i} \right)}^n}}}{{{{\left( {1+i} \right)}^n} - 1}}$$

where, *i* and *n* denotes 10% interest and 10-year system lifespan^56^.

The salvage value (S) is considered as 20% of CC^56^:

14 $$S=0.2 \times CC$$

Its annualized value (ASV) is followed by^56^:

15 $$ASV=SSF \times S$$

The sinking fund factor (SFF) is determined by^56^:

16 $$SFF=\frac{i}{{{{\left( {1+i} \right)}^n} - 1}}$$

The annual maintenance cost (AMC) is assumed to be 15% of FAC^56^:

17 $$AMC=0.15 \times FAC$$

Hence, the annual cost (AC) is estimated as^56^:

18 $$AC=FAC+AMC - ASV$$

The CPL and PBP are estimated by^56^:

19 $$CPL=\frac{{AC}}{{{P_d}}}$$

20 $$PBP=\frac{{Investments}}{{Net~earnings}}$$

The annual productivity ($${P_d})$$ is calculated for about 270 bright days. The profit-cost ratio (PCR) and cost of benefit (UAB) is evaluated by^57^:

21 $$PCR=\frac{{UAB}}{{AC}}$$

22 $$UAB={m_w} \times POW$$

Here, POW is considered as 0.1 $/L and the PCR value greater than 1 is required for the capital investment to be considered economically viable.

### Enviro-economic analysis

The enviro-economic assessment estimates CO₂ reduction and CCE for RDSS and DSS. Coal-fired power plants release 1.58 kg of CO₂ per kWh, which serves as the basis for comparison. The solar stills with 10-year embodied energy intake and production are considered in the environmental evaluation. The PV panels typically last 20–25 years, although the 10-year baseline is cautious and comparable which consistent with other studies^58^. The embodied energy input ($${E_{in}})~$$is the total energy required by system components during their life cycle, including raw material extraction, production, transportation, and assembly. The projected cost-based embodied energy input for RDSS and DSS is estimated as^59^:

23 $$~{E_{in}}=\frac{{CC}}{{0.14}}$$

The embodied energy output ($${E_{out}})~$$is calculated by^56^:

24 $$~{E_{out}}=\frac{{{m_w}~ \times ~L}}{{3600}}$$

The net CO_2_ mitigation ($${N_{c{o_2}}})~$$over the 10 years of lifetime ($$LT)~$$in RDSS and DSS is proposed by^56^:

25 $${N_{c{o_2}}}=\frac{{\left( {~{E_{out}} \times LT - {E_{in}}} \right) \times 1.58}}{{1000}}$$

The estimation of CCE in RDSS and DSS is given by^56^:

26 $$CCE={N_{c{o_2}}} \times {R_{c{o_2}}}$$

Here, $${R_{c{o_2}}}$$ denotes the international carbon cost of 14.5 $/ton of CO_2_ emission.

### Sustainability index

System sustainability is measured by energy conversion efficiency using the Sustainability Index (SI), which considered as a dimensionless metric. From exergy efficiency, it shows the way solar irradiation harvests energy while lowering irreversibility. The usual SI formula is determined by^51^:

27 $${\mathrm{SI}}={\mathrm{~}}\frac{1}{{1{ - _{{\mathrm{o}} - {\mathrm{ex}}}}}}~$$

**Fig. 4:**
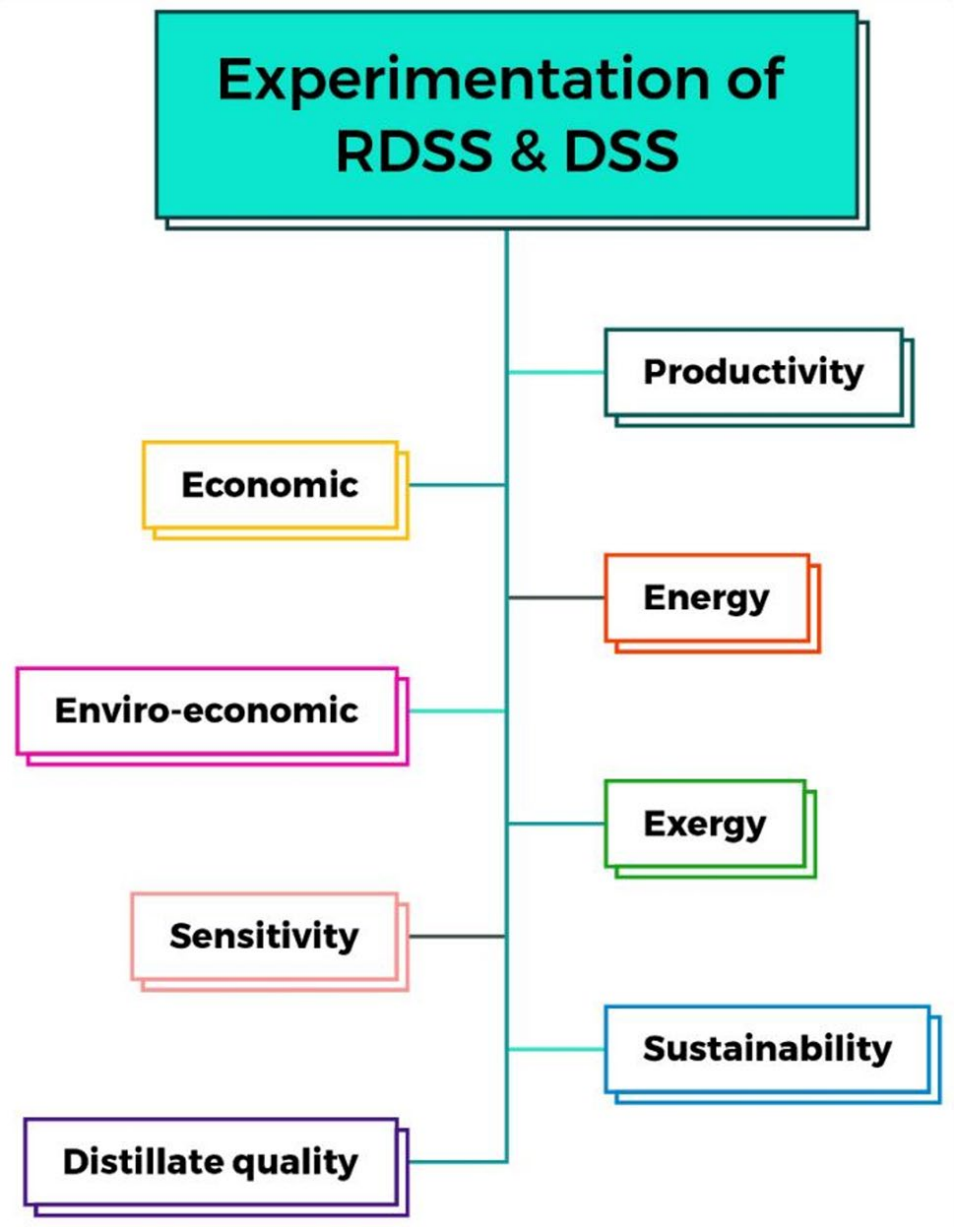
Experiment analyses done in RDSS and DSS.

Figure 4 shows the RDSS and DSS experiments analyses.

## Results and discussions

This section discusses and explains the RDSS and DSS performance assessment results which including productivity, energy and exergy performance, economic and environmental implications, sensitivity analysis, sustainability and distillate quality.

### Ambient conditions

**Fig. 5:**
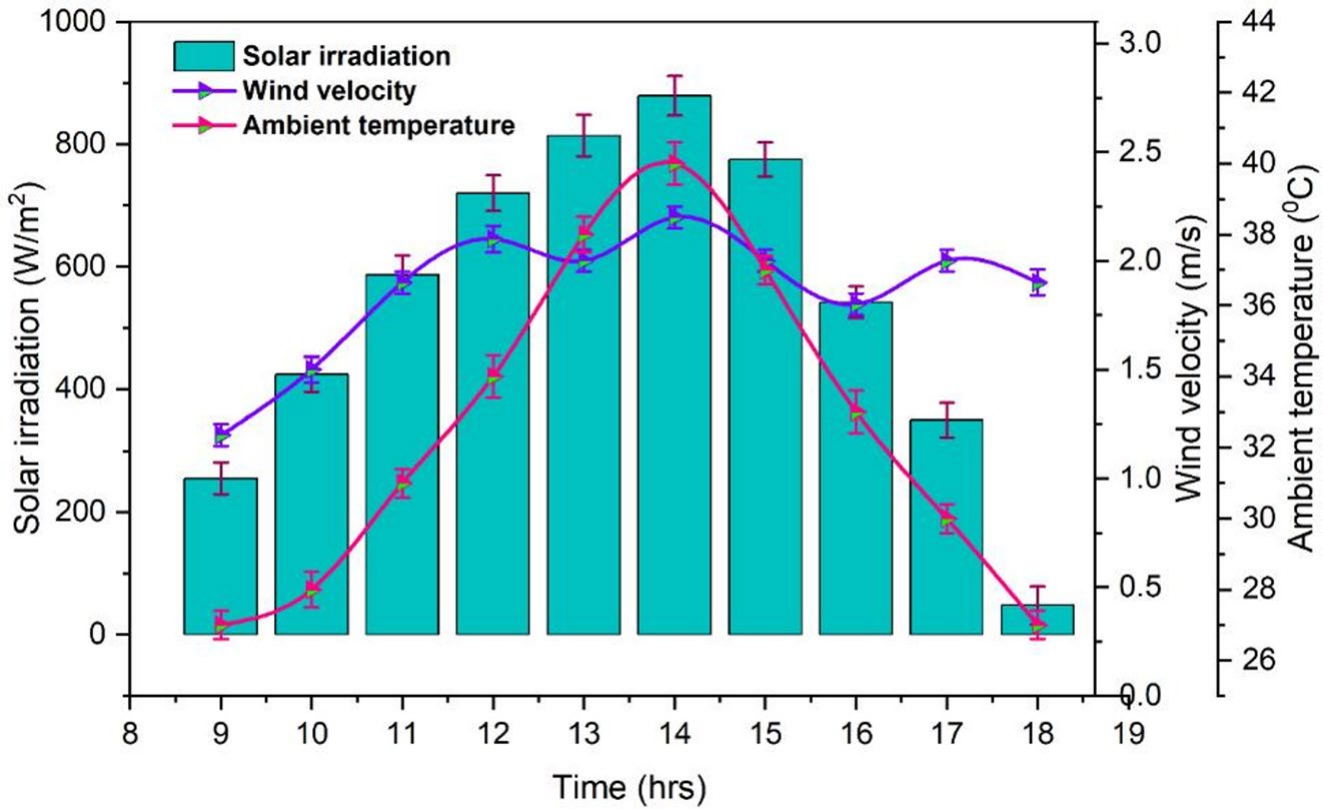
Fluctuations of solar irradiation and wind velocity with ambient temperature.

The fluctuations in solar irradiation, wind velocity and ambient temperature that were observed during the experimental operation of RDSS and DSS are depicted in Fig. 5. Throughout the day, the variations in wind velocity were noted with an average of 1.2 to 2.2 m/s. Because wind currents lowered the glass cover heat which importantly enhance the distillate. The pattern of ambient temperature followed that of solar irradiation which rising to over 39.9 °C at 14:00 h and falling to about 27.8 °C by sunset. Moreover, the increasing solar irradiation not only raised the ambient temperature but also enhanced PV power, whereas moderate to higher wind velocity enhance the distillate by facilitating heat at the surface of glass cover^60,61^. The intensity of higher solar irradiation was observed at 879.3 W/m² at midday and then steadily decreased to roughly 48.7 W/m² at 18:00 h. Even though sunlight was present for about 12 h, the systems only made optimal use of it for 8 to 10 h.

To quantify the percentage decrease in solar irradiation delivered into RDSS as a result of the PV-induced shaded region, the shadowing analysis was carried out. It was estimated considering the RDSS geometric dimensions and the solar-altitude parameters appropriate to the experimental location. The shadow length ($${L_{sh}})~$$of the solar PV panel is estimated using^62^:

28 $${L_{sh}}=\frac{{{\mathrm{h}} \times {\mathrm{sin~\boldsymbol{\uptheta}}}}}{{tan~\alpha }}$$

where, height of PV panel is $${\mathrm{h}}$$ (m). Additionally, $${\mathrm{\boldsymbol{\uptheta}}}$$ and $$\alpha$$ is title angle of PV panel and solar elevation angle (°). The shaded area ($${A_{sh}})~$$of the solar PV panel is observed by:

29 $${A_{sh}}=w \times {L_{sh}}$$

where, width of the PV panel is *w* (m). The shaded fraction ($${f_{sh}})$$ is calculated by^63^:

30 $${f_{sh}}=\frac{{{A_{sh}}}}{{{A_{DSS}}}}$$

where, $${A_{sh}}$$ is the area of the shadow region in m^2^. Finally, the radiation loss ($${R_{loss}})~$$is estimated by^64^:

31 $${R_{loss}}={f_{dir}} \times {f_{sh}}$$

where, ${f_{dir}}$ and ${f_{sh}}$ are the fraction of direct beam and shadow in %.

**Fig. 6:**
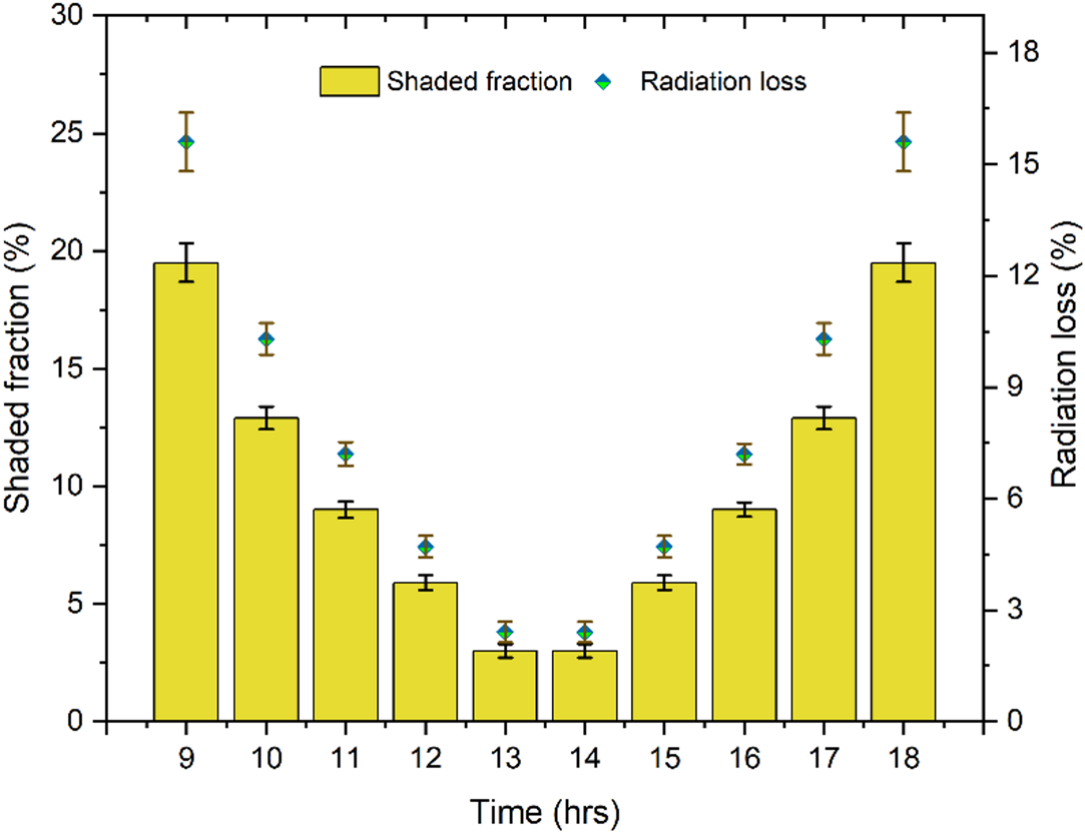
Variations of shadowing effect in RDSS experimentation.

The hourly shadowed fraction and radiation-loss on the RDSS glass cover are shown in Fig. 6. The trends match the daily solar altitude angle change. Due to low solar altitude, the PV panel creates the longest shadow at 09:00 and 18:00 h. The highest shaded fraction of 19.5% reduces effective transmitted radiation by 15.6%. With the sun rising between 10:00 and 11:00 h, the shadowed effect drops from 12.9% to 9%, resulting in 10.3–7.2% radiation losses. Furthermore, the sun reaches its maximum elevation at solar noon (12:00–14:00 h), which causes the shadow to greatly diminish. Similarly, the radiation loss was very low (2.4–4.7%), while the shadowed percentage falls to 3–5.9%. Since this time frame coincides with the most effective solar input, PV-induced shadowing has very little effect during hours of peak irradiation. Following 15:00 h, the trend symmetrically reverses as solar altitude falls, leading to a rise in the shadowed fraction and radiation loss towards 12.9% and 10.3% at 17:00 h, followed by another high at 19.5% and 15.6% at 18:00 h. Overall, the findings indicated that PV shading has the greatest effect on the system during off-peak hours which resulting in daily radiation losses that vary by time of day and typically range from 2 to 16%. Therefore, although PV-induced shading is unavoidable, its impact is largely confined to off-peak periods and does not compromise the overall thermal and productivity performance of the RDSS.

**Fig. 7:**
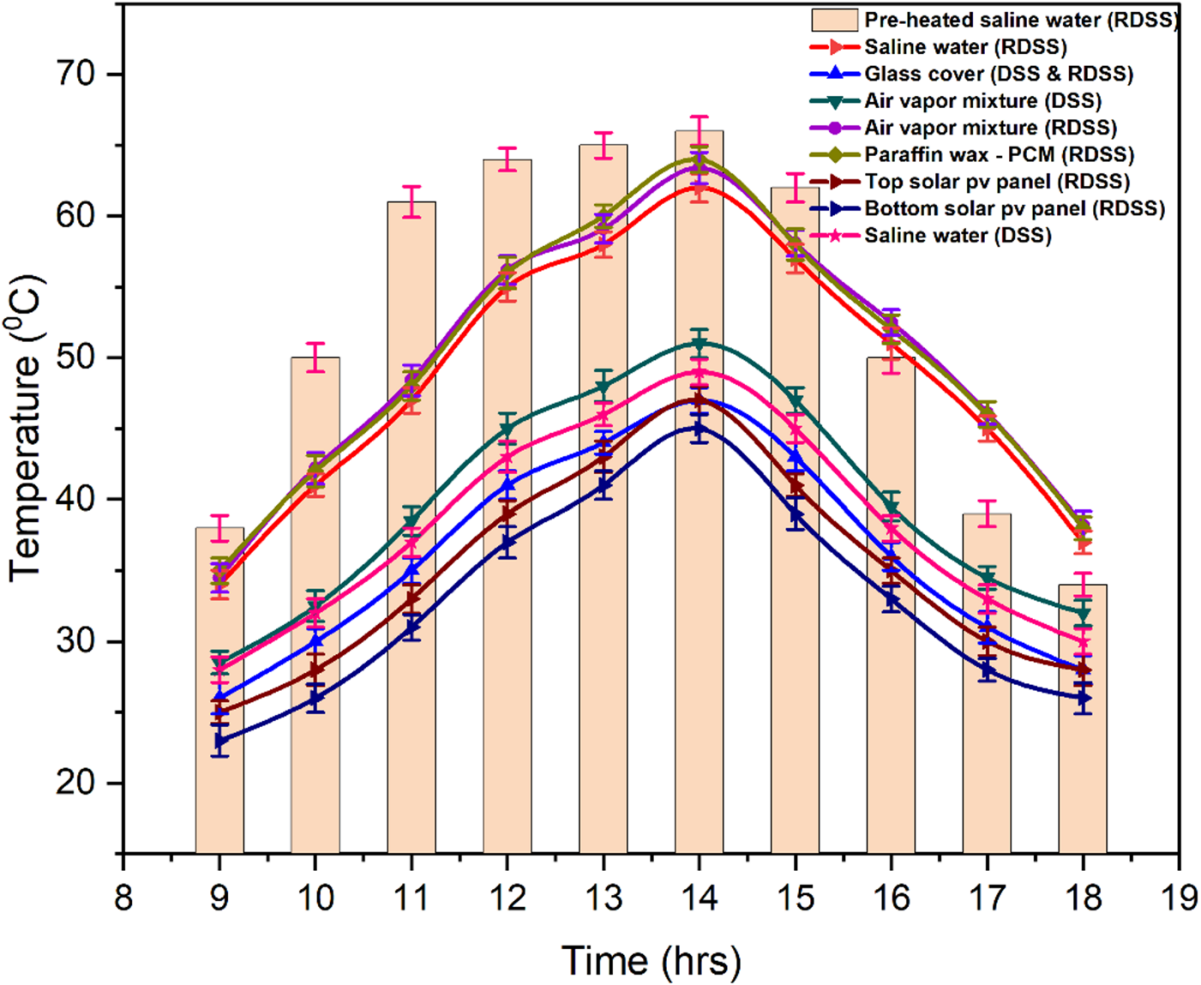
Effect of different temperatures in RDSS and DSS.

The effect of the various temperature profiles seen in RDSS and DSS is shown in Fig. 7. The glass cover is the best at using solar heat to evaporate saline water. Around noon, its temperature reached a peak of around 47.3 °C, and by the evening, it had progressively dropped to 27.7 °C. Because it was directly exposed to more solar flux, the upper surface of the solar PV panel continuously showed higher temperatures than the lower one. The PV power generation and solar irradiation were directly correlated, however PV efficiency declined as panel temperature rose. This emphasizes the importance to keep PV panel surfaces at their ideal working temperatures. The top and bottom PV panel recorded surface temperatures in RDSS were 47.1 °C and 44.8 °C, respectively, with a 5.1% discrepancy. The partial shadowing effort was produced by this variance which affected overall output. A heating element driven by the solar PV panel was used to provide preheated saline water to the RDSS basin in order to mitigate this impact. This feedwater temperature peaked at 66.2 °C at 14:00 h due to solar irradiation and PV output.

Adding paraffin wax PCM improved RDSS temperature response. The PCM temperature reached 63.8 °C at 14:00 h, when solar irradiation peaked. As solar input dropped, the PCM slowly released heat it absorbed under high irradiation. This helped RDSS when water and vapor temperatures dropped slower in the afternoon. RDSS maintained higher temperatures after 16:00 h due to PCM latent heat, while DSS showed a greater thermal decline. With less solar input, the PCM temperature dropped by the evening to values between 46.2 and 37.7 °C. By maintaining distillation during lower-solar irradiance hours, this prolonged release of stored heat significantly improved the system overall operational efficiency. The preheated feedwater caused the RDSS to achieve the air-vapor mixture temperature of 63.4 °C whereas the DSS only recorded 50.7 °C. This is an approximate 25.1% increase. Similarly, the RDSS saline water temperature peaked at 62.4 °C, while DSS was 48.9 °C which suggesting an approximately 27.6% increase in thermal storage capacity. The bottom PV panel also reduced shadowing by maintaining an optimal temperature gradient among the glass cover and the basin water, which promoted condensation. The controlled temperature at bottom side of PV panel and preheated inlet water and thermal capacity of PCM significantly decreased shadowing effect in RDSS and were major factors in its higher productivity compared to DSS.

### Productivity outcome

**Fig. 8:**
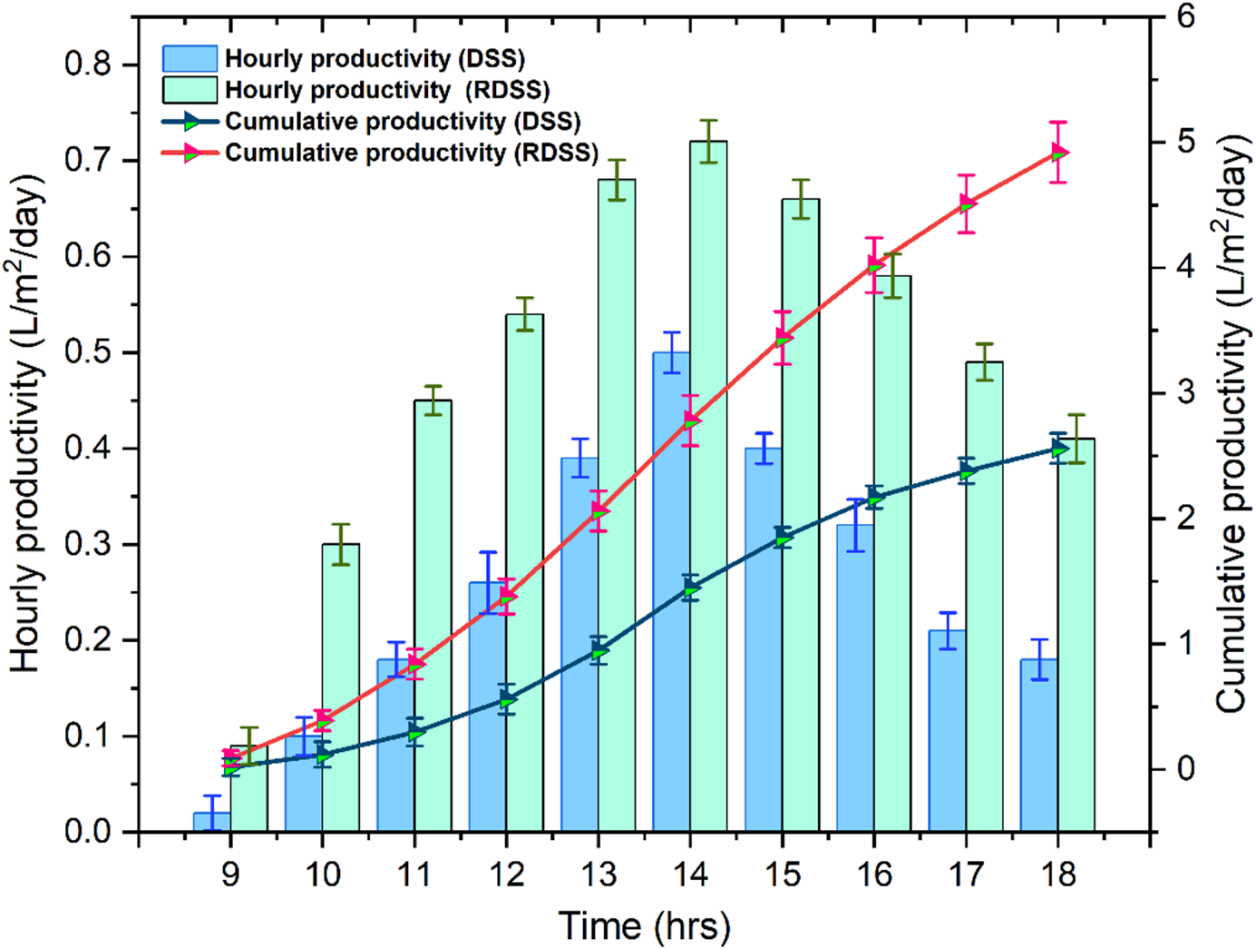
Variations of hourly and cumulative productivities in RDSS and DSS.

The hourly and cumulative productivities of RDSS and DSS are shown in Fig. 8. The RDSS had localized decreases in saline water temperature and evaporation as a result of the solar PV panel shade, which marginally decreased the basin exposure to sunlight. The basin temperature distribution became uneven as a result of this shading effect, which might have a slight impact on system performance as a whole. However, the both DSS and RDSS showed distinct reactions to changes in solar irradiation with productivity rising around noon at greatest sunlight and falling in the late afternoon. The hourly productivity in RDSS was about 0.7 L at 14:00 h, while DSS generated 0.5 L, which is a 46% increase in RDSS. The shadowing impact on surrounding areas generated a colder surface that enabled some condensation to remain, although the greater bottom PV panel temperature in RDSS slightly decreased condensation on the neighboring glass cover. Concurrently, the radiation and convection carried some heat from the PV panel to the basin which raising the temperature of the air-vapor mixture and increasing the rate of evaporation even in areas that were shaded locally. The higher evaporation rates which are essential for the production of freshwater were supported by the PV panel preheating of the saline water in conjunction with an electrical heating element.

Moreover, the thermal control was greatly aided by the addition of paraffin wax PCM. The PCM served as a thermal reservoir, absorbing excess heat during high solar irradiation periods and storing energy that could be released gradually as it dropped. The PCM temperature in RDSS peaked at 63.8 °C at approximately 14:00 h. In contrast to DSS, this heat storage allowed for a slower temperature drop by slowing down the basin late-afternoon cooling. Under reduced solar irradiation, the PCM temperature steadily dropped by the evening to values between 46.2 °C and 37.7 °C, permitting further evaporation during times when direct solar input was scarce.

The previously stored heat guarantees that the temperatures of the saline water stay high enough to support distillation, even though the PCM efficiency decreases at temperatures higher than 63.8 °C. Throughout the day, the RDSS continuously outperformed DSS, according to an analysis of hourly productivity profiles. The preheated saline water, bottom PV panel heat transfer, and PCM thermal storage worked in conjunction to improve evaporation which offset the little decrease in condensation brought on by shadowing effect. This resulted in a larger freshwater production in RDSS. The accumulation of moist air close to the glass cover, on the other hand, inhibited natural convection and decreased condensation efficiency, which is the main reason why DSS demonstrated lesser productivity. Cumulative productivity studies revealed RDSS surpassed DSS by 92.2%, producing 4.9 L/m²/day compared to 2.5 L/m²/day. This shows that preheating, PCM integration, and regulated PV panel shading reduced thermal losses, maintained higher basin temperatures, and promoted natural convection with improving system efficiency.

### Efficiency results

**Fig. 9:**
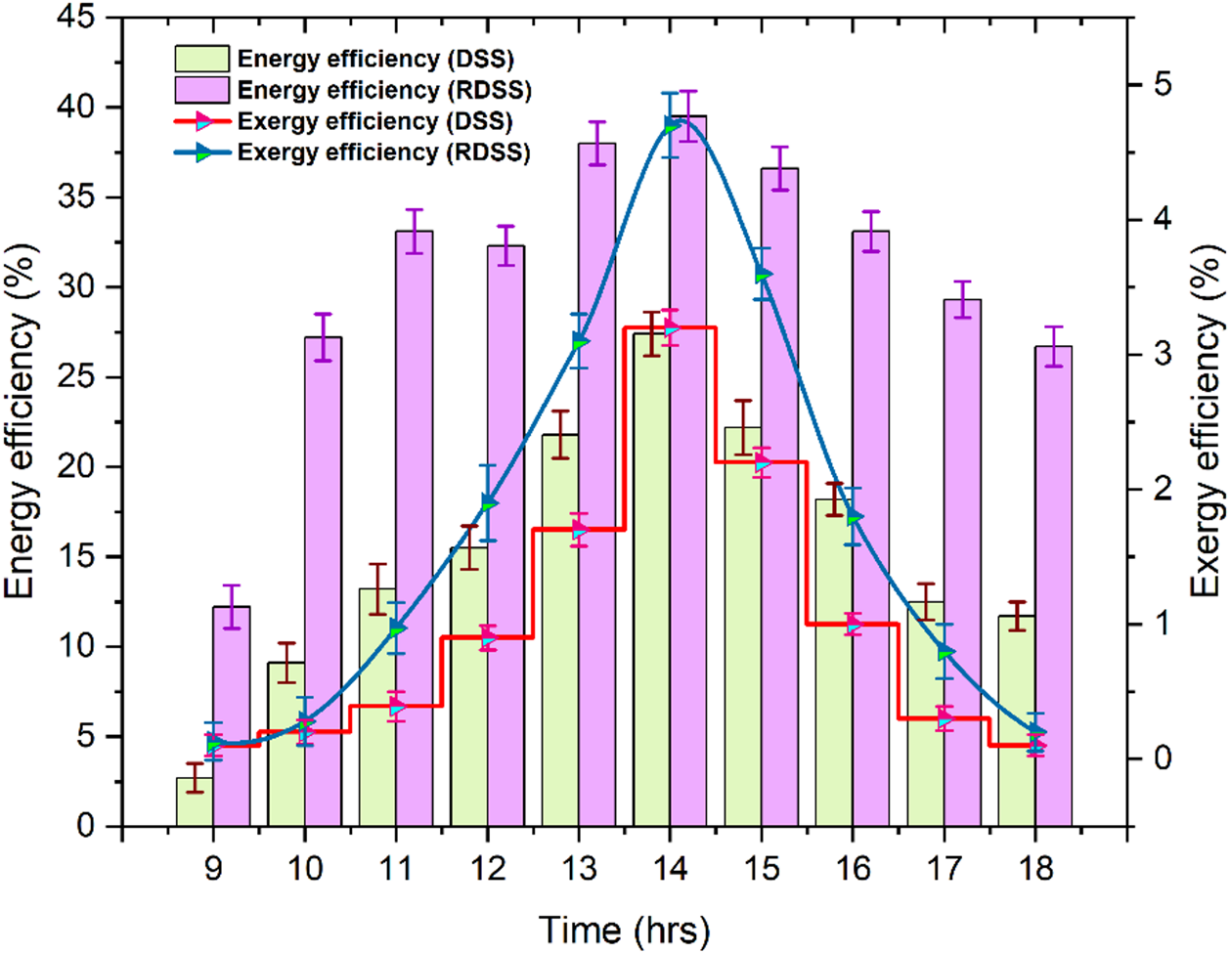
Impact of energy and exergy efficiencies in RDSS and DSS.

Figure 9 shows hourly RDSS and DSS energy and exergy efficiency effects. Similar to solar irradiation, both systems’ efficiency increased in the morning, peaked at 14:00 h, and decreased during nightfall. Energy efficiency was 44.2% higher in RDSS at 39.5% than DSS which had only 27.4%. The DSS exergy efficiency was 4.7% and 3.2%, whereas RDSS was 46.9% better. The PV-powered preheating, electrical heating element, and paraffin wax PCM thermal storage increase RDSS energy efficiency. At peak solar irradiation, the PCM accumulates heat and releases it gradually during evening time which supporting evaporation. Furthermore, the preheating saline water increases basin evaporation. Reduce energy losses and prolong evaporation by slowing basin temperature decrease and maintaining a more uniform thermal profile with latent heat storage. Energy efficiency shows the system’s ability to use energy efficiently and not irreversibly^65^. A lot of energy is lost in DSS due to poor heat transfer, humid air near the glass cover, and natural convection restrictions. The RDSS, it gains from improved heat transfer between the PV panel, basin water, and PCM which raises the temperature of the air-vapor combination and keeps a temperature gradient that is conducive to ongoing condensation. From an energy standpoint, the RDSS is a more efficient system because of its optimized energy flow which lowers thermodynamic losses. All things considered, these enhancements demonstrate that RDSS is a resource-efficient and sustainable method of producing freshwater. In addition to increasing productivity, the RDSS uses solar energy thermodynamically better by combining preheating with PCM-based thermal storage, which helps to mitigate water scarcity and meet energy conservation goals.

**Fig. 10:**
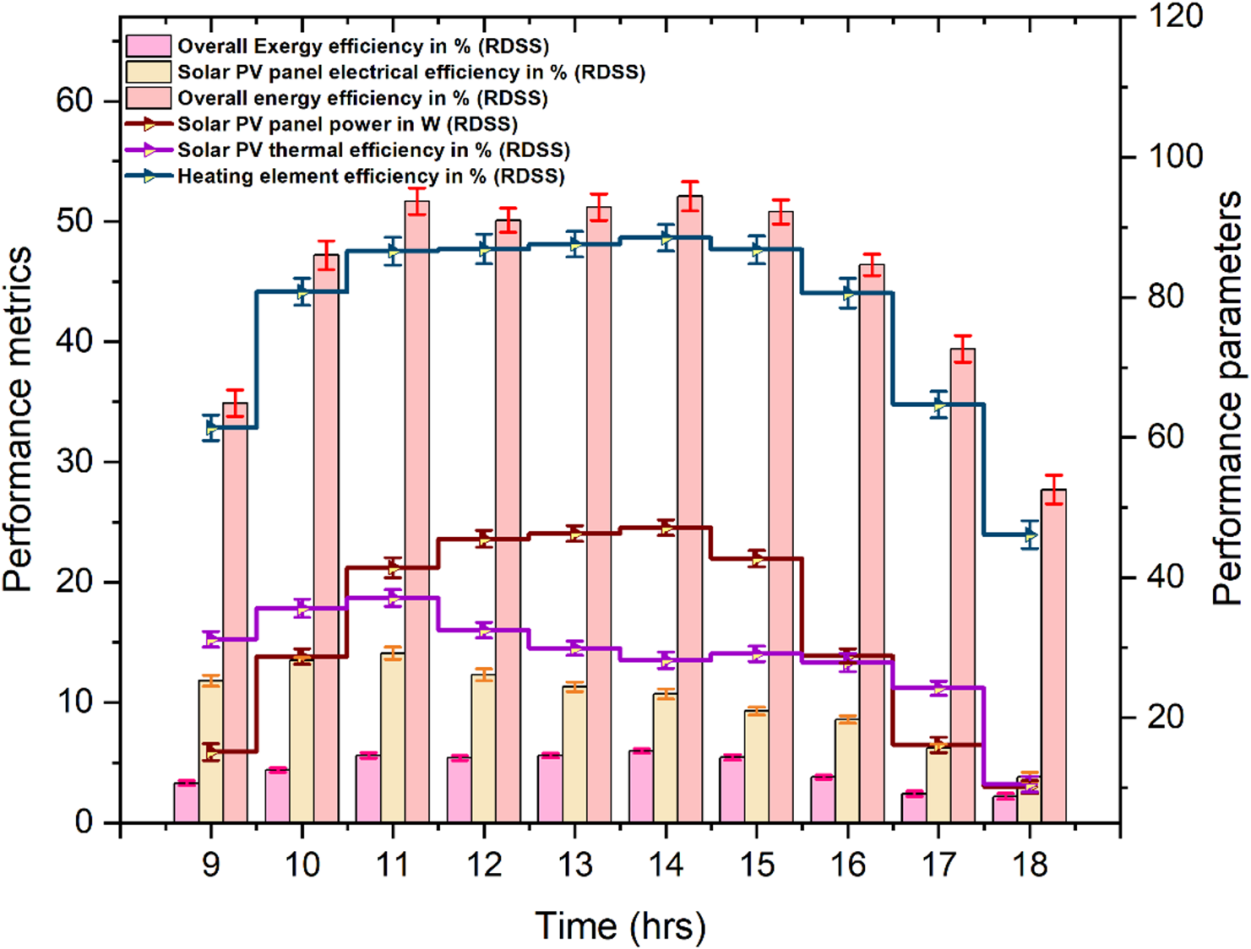
Impact of different performance metrics in RDSS.

The power output, overall energy and exergy efficiencies, electrical and thermal efficiencies and heating element performance of RDSS are shown in Fig. 10. The heating element used to prepare the saline water before it entered the RDSS basin was powered by the solar PV panel which peaked at 47.1 W at 14:00 h. This resulted in a maximum preheated water temperature of roughly 66.2 °C. At 17:00 h, when the saline water was warmed to 38.9 °C, the lowest power output of 16.1 W was measured. The solar PV panel produced a mean warmed water temperature of roughly 52.9 °C while delivering an average of 32.1 W of power. By using PV-generated electricity to offset shadowing effects and maintain basin temperature, the electrical heating element incorporation improved the RDSS performance. At 11:00 h, the system maximum electrical and thermal efficiencies were observed to be around 14.1% and 37.1%, respectively. The hourly productivity, PV power output, solar irradiation, ambient temperature, and energy and exergy efficiency all reached their peak performance at 14:00 h. Thermal losses brought on by the high temperatures somewhat decreased overall efficiency even if the PV panel produced more absolute power during this period. The decline in open-circuit voltage causes RDSS electrical and thermal inefficiencies at higher PV panel temperatures which impairs system performance as a whole. The heating element efficiency peaked at 88.5% at 14:00 h, which also occurred to be the maximum PV power output. This shows that the heating element power and heat supply are directly correlated with the energy generated by the PV panel.

The sum of RDSS electrical, thermal, and heating element contributions were used to determine its overall energy efficiency. It was an average of about 45.1%, with the maximum figure of 52.1% occurring during 14:00 h. Throughout the day, there were a few minor temporal fluctuations, such as 51.7% at 11:00 h, 51.2% at 13:00 h, and a slight decline to 50.1% at 12:00 h. These variations are a result of increased thermal losses and a modest decrease in the PV panel electrical efficiency caused by the higher noon solar irradiation which also affected the instantaneous energy conversion. At 14:00 h, the total exergy efficiency of RDSS peaked at 5.9% which implying fewer irreversibility along with more thermodynamically efficient solar energy absorption. The system reduces energy waste and improves the conversion of solar input into productive activity for both electricity generation and water evaporation by efficiently storing and using thermal energy. Another benefit of the suggested design is that, in case that the heating element is not used, the average electrical power generated by the PV panel could provide an additional benefit for family electricity use. In conclusion, the RDSS outperforms DSS in terms of freshwater productivity and energy and exergy performance. Combining preheating, PV-powered heating, and thermal storage via PCM enables more effective use of solar energy, lower thermal losses, and continuous operation in a range of solar conditions. These characteristics contribute to long-term resource conservation and energy-efficient operation which making RDSS a viable and sustainable option for producing electricity and drinkable water simultaneously.

### Economic possibility

Tables 2 and 3 provide a summary of the economic viability of RDSS in comparison to DSS, emphasizing important financial factors. In this, the actual costs may differ between nations and over time as a result of market trends, however the cost analysis is predicated on the current study estimated pricing in India. The DSS estimated CC is about 94.78 $, whilst RDSS is about 148.95 $. A thorough assessment of economic feasibility is given in this section which showed that RDSS has greater long-term economic benefits than DSS, especially given its dual purpose of providing energy and freshwater. The CPL is one of the most significant economic measures linked to productivity^66^. The estimated CPLs for RDSS and DSS were about 0.019 $ and 0.023 $, respectively which showing that RDSS outperforms DSS in terms of production cost by 17.3%. This twin advantage of generating power and distilled water improves economic sustainability even more.

**Fig. 11:**
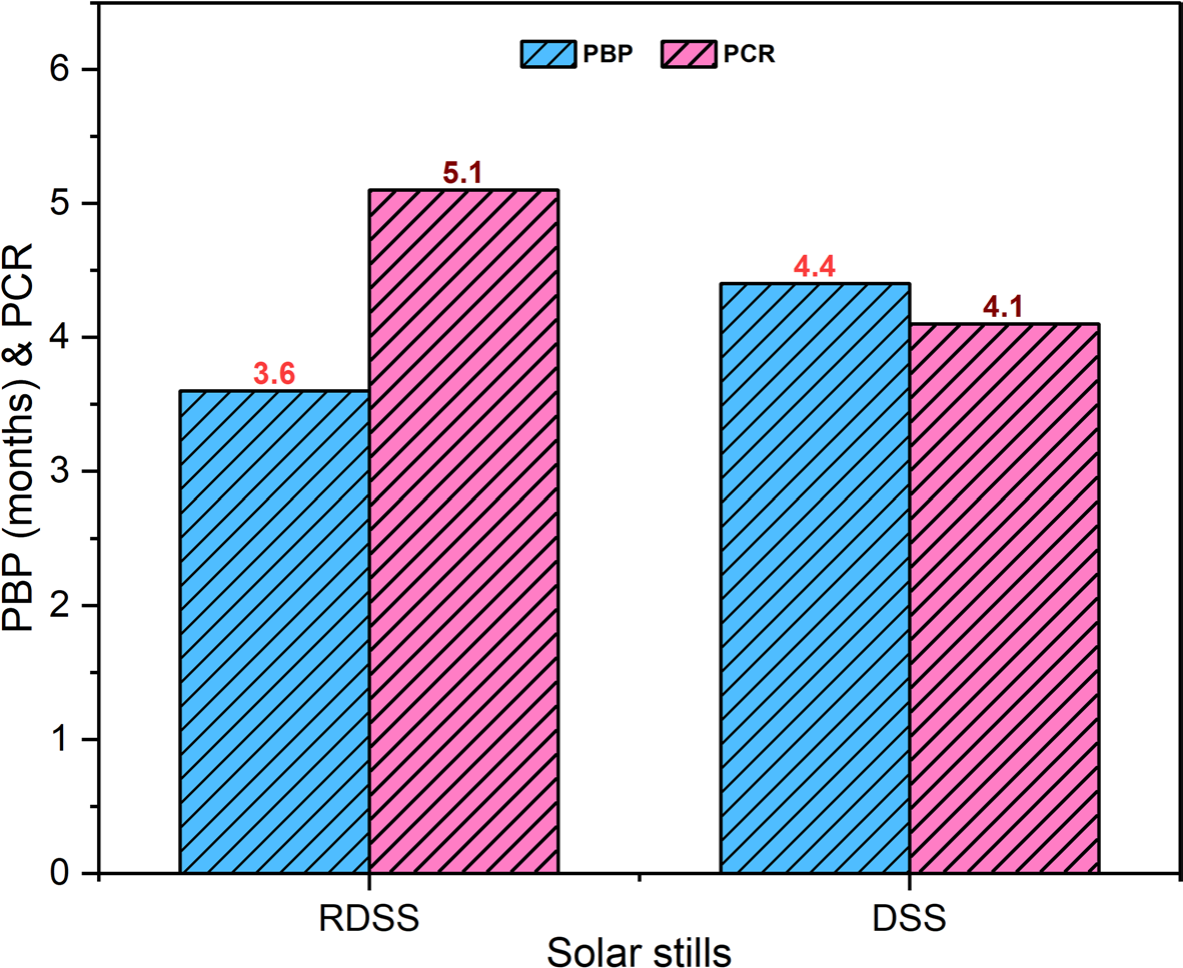
Estimation of PBP and PCR of RDSS and DSS.

Additional financial measures, such as PCR and PBP are shown in Fig. 11. The DSS needed roughly 4.4 months, however RDSS had only 3.6 months which indicating that RDSS has an 18.2% quicker PBP. The inexpensive PBP is due to inexpensive setup costs, passively functioning (no pumps or energy), and higher sunlight-powered distillate. The recovery of the primary cost at a few months is the emphasis of estimation of PBP even if PV panels have a lengthy operational life. The variations in PV panel pricing and maintenance requirements are the cause of minor PBP variances between RDSS and DSS. A more profitable operation was shown by the 19.6% higher PCR value that RDSS recorded than DSS. A PCR larger than one indicates that the system makes more money than it spends which providing a solid basis for long-term and sustainable functioning. These findings highlight the benefits of RDSS over traditional DSS in terms of economy and sustainability which makes it a viable choice for combined freshwater and electricity generation.

**Table 2:** Manufacturing costs of the RDSS and DSS.

Solar still elements	Cost ($)	
	DSS	RDSS
Basin layer	20.36	20.36
Build layer	33.45	33.45
Glass cover	23.87	23.87
Insulation	9.22	9.22
Basin layer paint	2.10	2.10
Rubber seal	5.78	5.78
Water tank	-	10.08
Paraffin wax	-	2.15
Heating element	-	6.65
PV panel	-	35.29
Total	94.78	148.95

**Table 3:** Economic analysis of the RDSS and DSS.

Parameters/ solar stills	CC ($)	AMC ($)	AC ($)	m_w_ (L/year)	CPL ($)
RDSS	148.95	3.6	25.9	1328.4	0.019
DSS	94.78	2.3	16.4	691.2	0.023

#### Economic sensitivity analysis

**Fig. 12:**
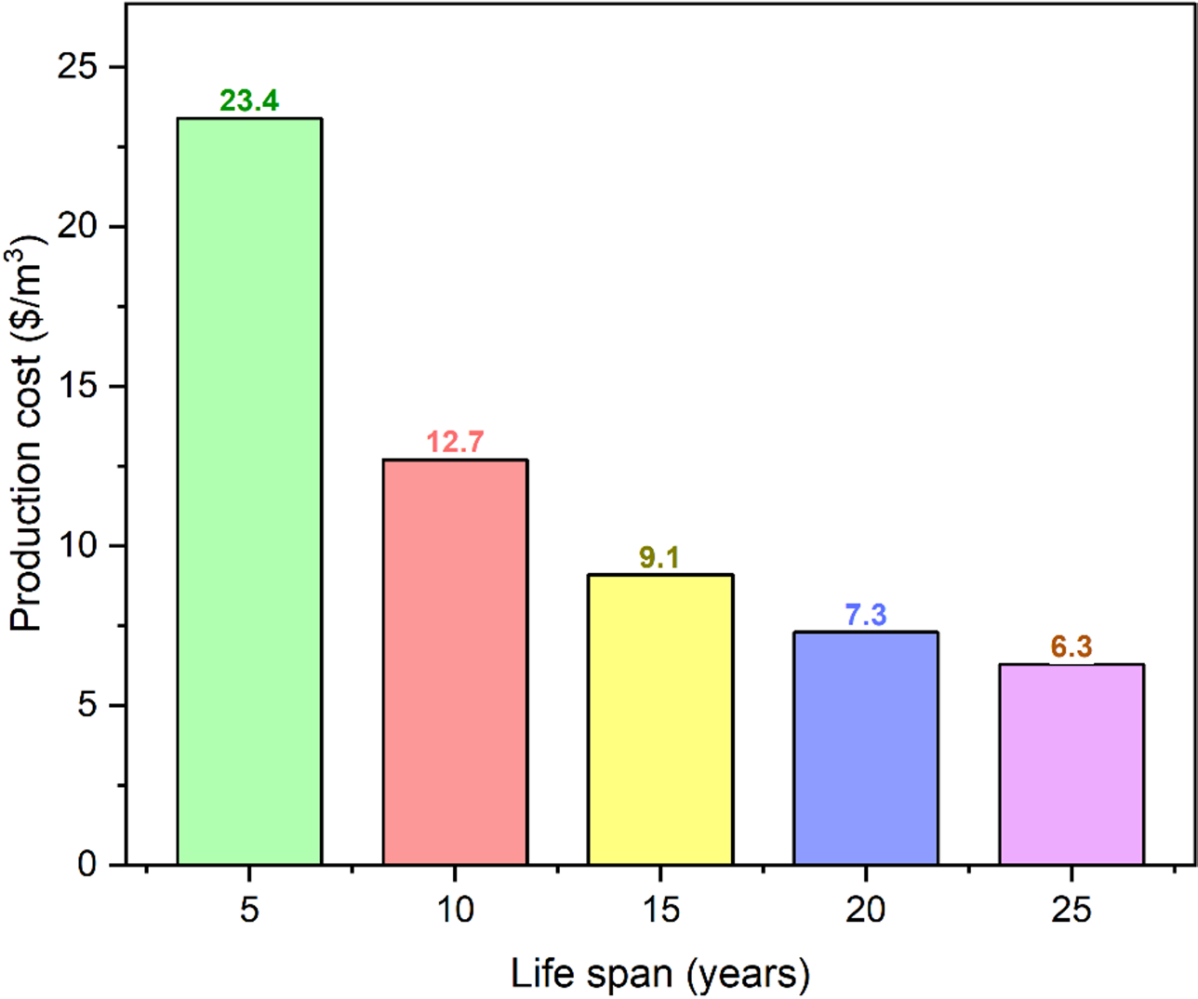
Estimation of production cost with lifespan.

The impact of interest rate fluctuations and the RDSS operating lifespan on the production cost was evaluated by a thorough sensitivity analysis which offered important fresh perspective on the main determinants of long-term economic viability. System design optimization and financial planning using sensitivity analysis ensure technological viability in diverse deployment scenarios and economic situations. RDSS operating lifespan can lower production cost by spreading out the initial capital investment over time. At a fixed interest rate of 2.5%, manufacturing expenses decreased by 73.1% from 23.4 $/m^3^ for a 5-year lifespan to 6.3 $/m^3^ for a 25-year lifespan as illustrated in Fig. 12. Durability is crucial to decreasing long-term expenses because lifetime and production cost are negatively correlated across all interest rates. These data showed that RDSS operating life requires strong design and high-quality materials. With low-interest financing, 20–25-year lifespan systems can reduce production costs to below $10/m³, enhancing their cost-effectiveness. This analysis reveals those intentional investments in system longevity and reliability boost RDSS sustainability and appeal for large-scale, long-term water and energy production projects while minimizing economic risk.

**Fig. 13:**
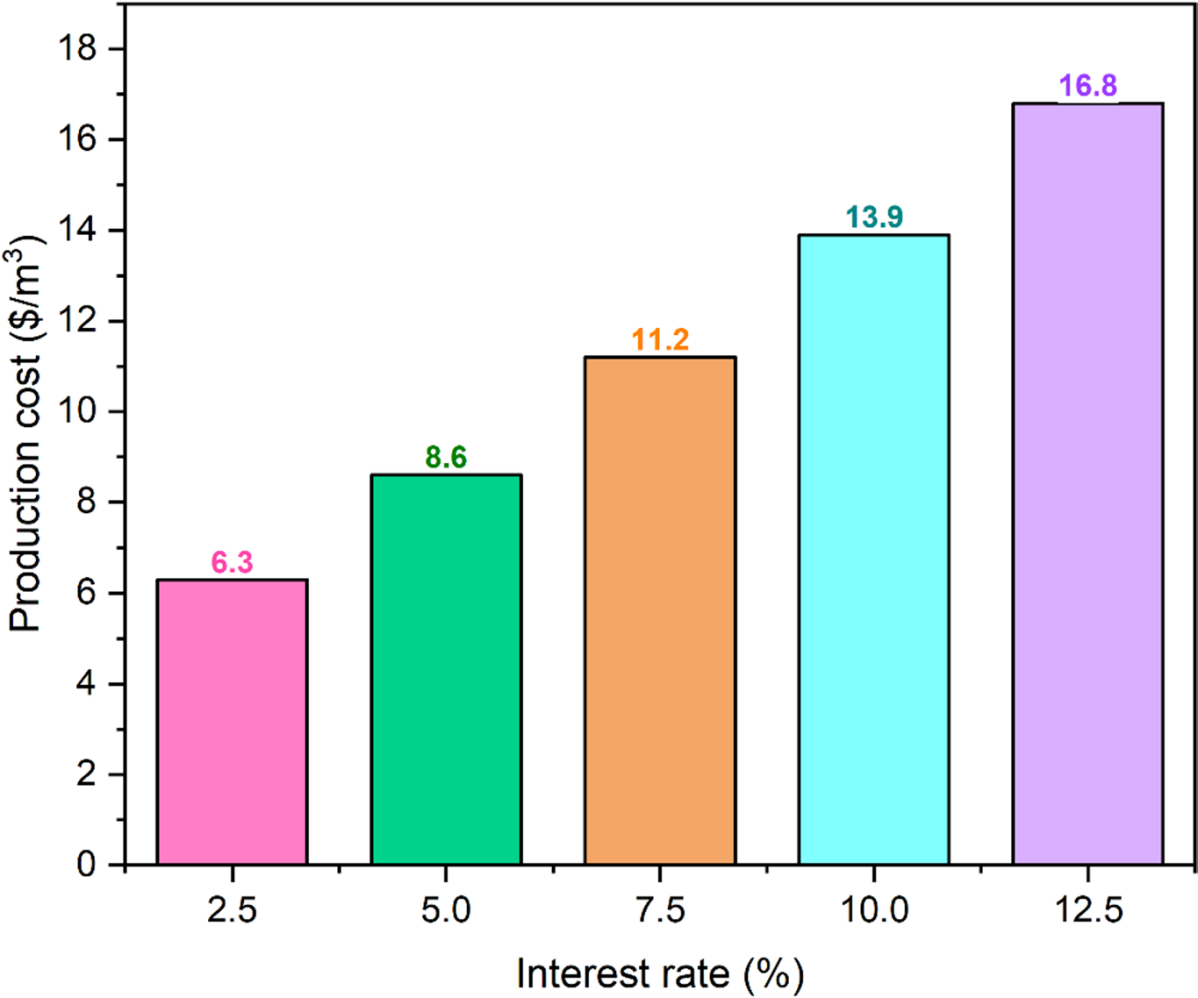
Estimation of production cost with interest rate.

Conversely, higher interest rates increase financial commitments and production expenses. Figure 13 shows that the production cost of a 25-year RDSS system rises by 166.6% from 6.3 $/m^3^ at 2.5% interest rate to 16.8 $/m^3^ at 12.5% interest rate. This high interest rate sensitivity highlights the importance of securing government-backed subsidies or concessional loans to mitigate high CC. In locations without accessible finance, RDSS economic feasibility may suffer. Shorter-lived RDSS systems are more susceptible to interest rate swings, according to statistics. Due to increased baseline production costs, financing conditions affect overall cost more. For example, a 5-year system production cost rises from $23.4/m^3^ at 2.5% to $32.7/m^3^ at 12.5%. In contrast, a 25-year system shows a growth from 6.3 to 16.8 $/m^3^ in the same interest rate range. The shorter-lived systems are more vulnerable to financial volatility, as shown by their larger relative impact of interest rate changes, even when they have a higher absolute cost increase.

In order to improve economic stability, these findings highlight the importance to design RDSS for longer operational lifespans and to get advantageous financing terms. Table 4 summarizes the results of sensitivity analysis on how interest rates and longevity affect manufacturing costs. It showed that extending the system operating life and ensuring low-interest financing are key to increasing solar still technology financial feasibility. Sensitivity data showed that a 25-year RDSS with a 2.5% interest rate has a production cost of 6.3 $/m^3^ which is significantly lower than a 10-year system with a 10% interest rate (19.5 $/m^3^). Quality materials and design must be used to ensure system durability to save money over time. To decrease CC’s impact on end users, regulators can give lower interest rates or other financial incentives. These findings support larger techno-economic assessments of solar distillation systems that highlight creative designs for sustainable freshwater production. RDSS’s increased performance and economic parameters strengthen its potential for large-scale deployment, especially in water-stressed nations, with long-term durability and supporting financial plans.

**Table 4:** Sensitivity analysis of production cost for varying lifespan and interest rate.

Lifespan (years)		5	10	15	20	25
Interest rate (%)	2.5	23.4	12.7	9.1	7.3	6.3
	5	25.7	14.9	11.3	9.6	8.6
	7.5	28.1	17.2	13.7	12.1	11.2
	10	30.3	19.5	16.2	14.7	13.9
	12.5	32.7	22.1	18.8	17.5	16.8

### Environmental impact

**Fig. 14:**
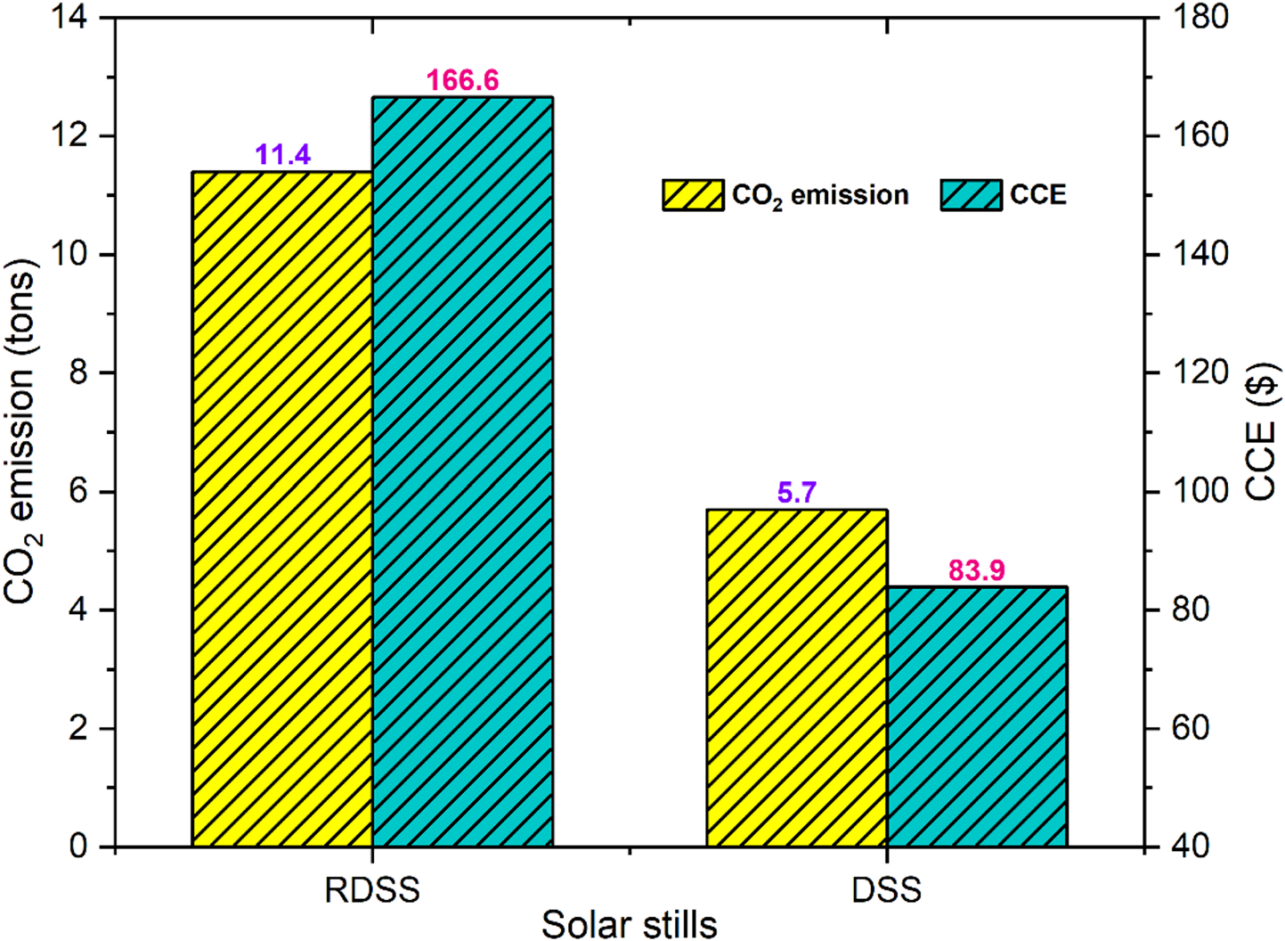
Estimation of CO_2_ mitigation and CCE in RDSS and DSS.

The study of embodied energy across various solar still components emphasize the relevance of sustainability and environmental performance^67,68^. Table 5 shows the estimated embodied energy for RDSS and DSS at 1063.8 and 676.9 kWh, respectively. This suggests that RDSS has a higher initial energy expenditure due to its more integrated components. Table 6 compiles the associated enviro-economic parameters, providing a comprehensive knowledge of the system’s financial and environmental performance throughout its operation. In Fig. 14, RDSS and DSS are expected to accomplish 11.3 and 5.7 tons of CO_2_ emissions over a ten-year period, respectively, implying that RDSS delivers a net CO_2_ reduction that is approximately 98.2% more than DSS. The combined solar PV panel, electrical heating element, and PCM work together to improve total thermal efficiency and productivity which resulting in improved performance. The estimated CCE values for RDSS and DSS are 166.6 and 83.9 $, respectively. It reveals that higher production is favorably related to both CO_2_ reduction and CCE. These findings demonstrated that the RDSS works to enhance energy efficiency while minimizing environmental effect. Furthermore, the results indicate that even though RDSS has a greater embodied energy investment, its longer lifespan and better operating performance allow for significant financial and environmental benefits which eventually improve sustainability. All things considered, the RDSS exhibits a very positive balance between energy input, freshwater output, and environmental advantages which making it a solution that is both inexpensive and environmentally efficient. The system considerable potential for recovering carbon costs and supporting sustainable water production make it a good choice for long-term implementation in areas with limited energy and water resources.

**Table 5:** Embodied energy of the various solar still components^59^.

Solar still components	Embodied energy (kWh)	
	DSS	RDSS
Basin layer	145.4	145.4
Build layer	239	239
Glass cover	170.5	170.5
Insulation	65.8	65.8
Basin layer paint	15	15
Rubber seal	41.2	41.2
Water tank	-	72
Paraffin wax	-	15.3
Heating element	-	47.5
PV panel	-	252.1
Total	676.9	1063.8

**Table 6:** Enviro-economical parameters of the RDSS and DSS.

Solar stills	Annual productivity (L/year)	Embodied energy input (kWh)	Embodied energy output (kWh)
RDSS	1328.4	1063.8	833.9
DSS	691.2	676.9	433.9

#### Environmental sensitivity analysis

**Fig. 15:**
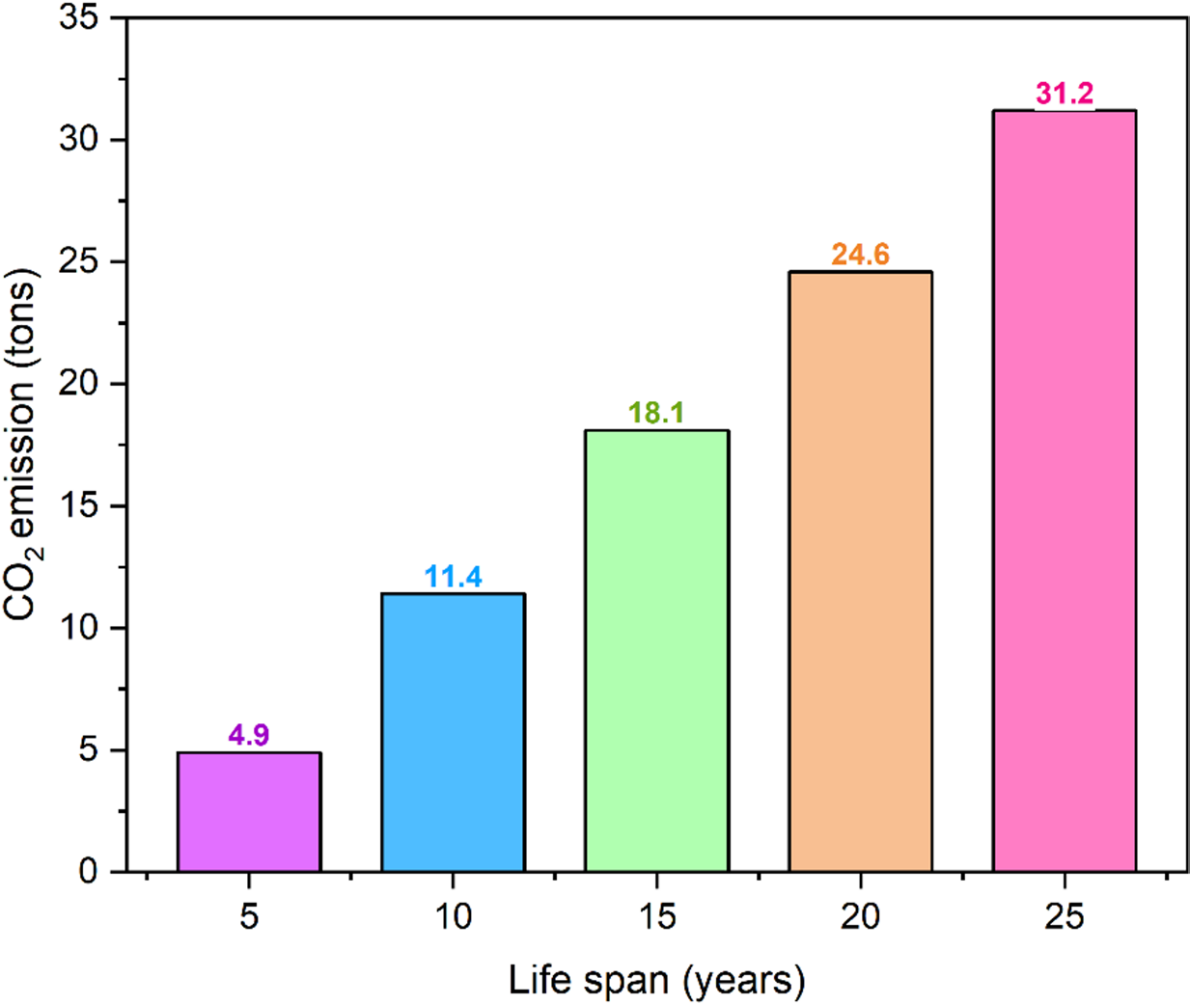
Variations of CO_2_ mitigation in RDSS with lifespan.

**Fig. 16:**
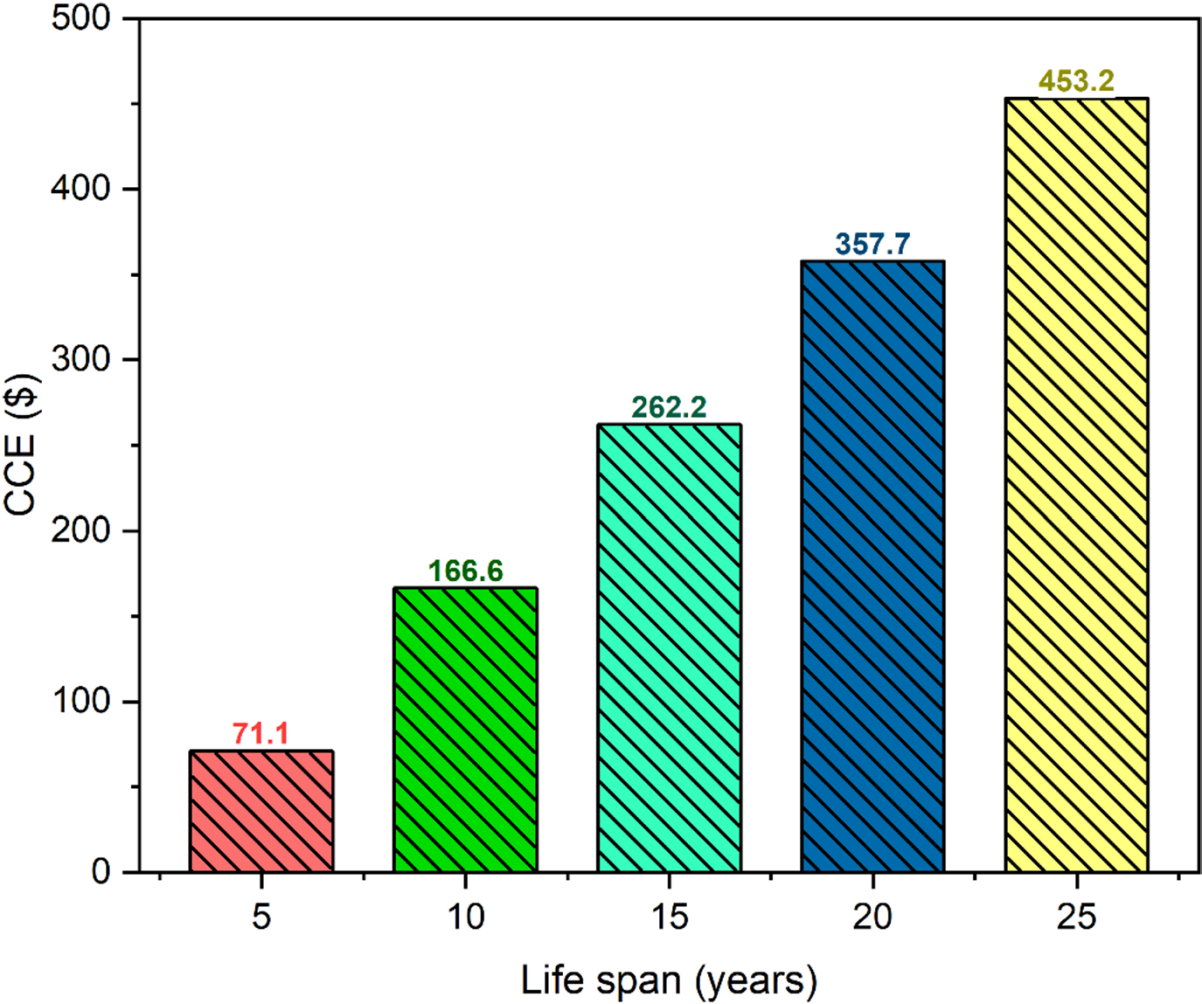
Variations of CCE in RDSS with lifespan.

A number of various operational lifespans were used to evaluate the environmental and enviro-economic performance of RDSS. The results showed that there were considerable gains in both the reduction of CO_2_ emissions and the financial advantages that are connected with them. As the lifespan of the system expands from 5 to 25 years, the total amount of CO_2_ that is reduced goes up from 4.9 ton to 31.2 ton which is a growth of 536.7% (see Fig. 15). At the same time, the economic value of the system goes up from 71.1 $ to 453.2 $ (see Fig. 16), which is a comparable percentage increase as 537.4%. This illustrates that there is a virtually linear association between the lifespan of the system and the cumulative environmental benefits with an average monetary value of approximately 18.1 $ per ton of CO_2_ that is mitigated. The consistent rise in CO_2_ reduction emphasizes the vital need of developing RDSS for long-term operation in order to maximize the benefits to the climate. Further improvements in thermal efficiency and durability can be achieved by enhancements in PCM selection, system design optimization and routine maintenance. This will result in an increase in the system overall impact on the environment over the course of its lifetime. These findings demonstrate the twin benefits of RDSS deployment in terms of policy and decision-making. The first advantage is long-term climate mitigation through significant CO_2_ reductions, while the second is predictable economic returns. By quantifying both absolute and percentage improvements, stakeholders can have a clear framework for evaluating the system’s sustainability, cost-effectiveness, and long-term environmental contributions. This enables intelligent planning for large-scale adoption in areas with water and energy stress.

### Sustainability analysis

**Fig. 17:**
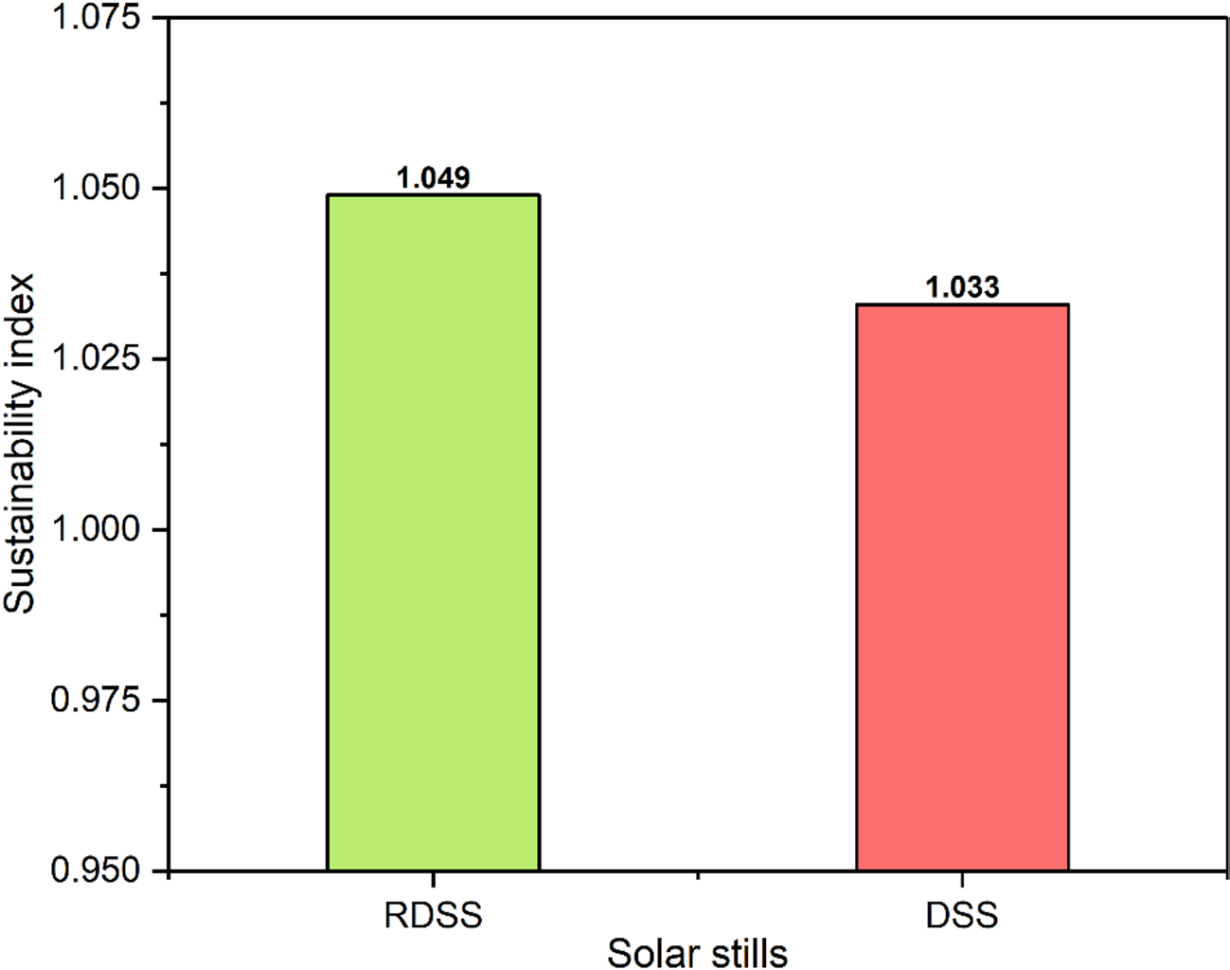
Sustainability index of RDSS and DSS.

Exergy destruction is used to assess energy degradation in a system, which is essential in terms of sustainability goals. The maximum SI values for the RDSS and DSS systems were 1.049 and 1.033, respectively, as shown in Fig. 17. The SI is a normalized composite statistic that measures simultaneous improvements in a variety of domains, including energy efficiency, exergy recovery, embodied-energy payback, environmental mitigation, and economic performance. The 1.6% numerical difference may appear minor, yet it has practical significance. The RDSS, with a 5.9% higher exergy efficiency and accordingly greater SI than the DSS, demonstrates that it can successfully gather and use solar energy to generate power and fresh water. The PV panel, electrical heating element, and PCM are integrated to produce this improved performance, which lowers energy losses and increases productivity, enhancing operational and environmental results. The traditional DSS, on the other hand, only generates freshwater, which restricts its sustainability and energy consumption. According to these results, multipurpose solar still designs that use thermal storage and energy recovery techniques can enhance system longevity, environmental performance, and energy efficiency^69^.

### Existing literature comparison

A comprehensive overview of DSS systems combined with solar PV panels, electric heaters, and PCM is given in Table 7, which is especially relevant to the current investigation. Combining PV panels with electric heaters enhanced freshwater productivity to 2.1–5.7 L/m^2^/day, according to Riahi et al.^70,71^. The studies were carried out over two to three days, with water depths of 3 to 4 cm. However, these studies limited a complete evaluation of their economic and environmental consequences by missing to report PCM mass and frequently lacking important performance indicators including energy and exergy efficiency, distillate cost, and CO_2_ mitigation potential. In a thorough performance evaluation of a DSS with PV and an electric heater, Dhivagar et al.^15^ achieved a productivity of 4.3 L/m^2^/day, energy and exergy efficiencies of 37.4% and 4.4%, a distillate cost of 0.024 $/L, and a CO₂ mitigation potential of 9.9 tons with a one-day experiment conducted at a water depth of 2 cm. In the current study, the suggested RDSS used 0.5 kg PCM to produce improved CO₂ mitigation of 11.4 tons, lower distillate cost of 0.021 $/L, energy and exergy efficiencies of 39.5% and 4.7%, and increased freshwater productivity of 4.9 L/m^2^/day. At a water depth of 2 cm, experiments were carried out for a single day under an average solar irradiation of 879.3 W/m^2^. Overall, the current RDSS is more cost-effective, sustainable, and balanced than earlier research, which makes it especially appropriate for areas with limited water resources. PCM enhances energy efficiency, environmental performance, and economic feasibility when combined with PV and electric heating.

.

**Table 7:** Comparison of the RDSS in existing literature.

Experimentation	Reconfiguration	Experiment days	Solar irradiation (W/m^2^)	Water depth (cm)	PCM mass (kg)	Distillate (L/m^2^/day)	Energy efficiency (%)	Exergy efficiency (%)	Distillate cost ($)	CO_2_ emissions (tons)
Riahi et al.^70^	DSS + PV panel + Electric heater	3	823	4	-	5.7	-	-	-	-
Riahi et al.^71^	DSS + PV panel + Electric heater + Sand	2	1078	3	-	2.1	-	-	-	-
Dhivagar et al.^15^	DSS + PV panel + Electric heater	1	840.1	2	-	4.3	37.4	4.4	0.024	9.9
Present study	DSS + PV panel + Electric heater + PCM	1	879.3	2	0.5	4.9	39.5	4.7	0.021	11.4

### Distillate analysis

Table 8 compares essential water quality indicators for saline water, RDSS and DSS to WHO drinking water criteria^72^. The water was unfit for human consumption due to high levels of TDS (1,000–4,000 ppm), chloride (200 − 20,000 mg/kg), electrical conductivity (1,500-5,000 µS/cm), salinity (35–40 ppt), and turbidity (6.5–8.5 pH). In this, the RDSS lowered TDS to 17 ppm, chloride to 2.3 mg/kg, electrical conductivity to 19 µS/cm, and salinity to 0.014 ppt, while DSS had slightly higher values of 22 ppm, 2.9 mg/kg chloride, 20 µS/cm conductivity, and 0.019 ppt salinity. Both systems met WHO criteria with 0.2 NTU turbidity and 6.5–6.6 pH. These data showed that RDSS removes contaminants better than DSS by yielding purer distillate. The preheating saline water and increasing energy input in RDSS improves evaporation and lowers residual dissolved salts. Finally, the RDSS and DSS both generate drinkable water, however the RDSS is better at purifying saline water.

**Table 8:** Distillate quality analysis.

Parameter	Saline water	RDSS	DSS	WHO standard
Total dissolved solids (ppm)	1,000–4,000	17	22	≤ 500
Chloride (mg/kg)	200 − 20,000	2.3	2.9	250
Electrical conductivity (µS/cm)	1,500-5,000	19	20	≤ 500
Turbidity (NTU)	High	0.2	0.2	≤ 0.5
Salinity (ppt)	35–40	0.014	0.019	≤ 0.5
pH	6.5–8.5	6.5	6.6	≤ 6.5–8.5

## Conclusions

The performance of RDSS and DSS was tested in similar climates. In this, the RDSS combined a solar PV panel with an electrical heating element with paraffin wax PCM to generate freshwater, thermal energy, and electricity. The paraffin wax PCM improved thermal storage and the PV panel shadowing effect was mitigated by powering the heating element to preheat the incoming saline water. From these observations, the following conclusions can be drawn:

1. In RDSS and DSS, the highest recorded saline water temperatures were roughly at 62.4 °C and 48.9 °C, respectively. The preheating and the addition of PCM together produced the RDSS a 27.6% greater capacity for heat storage than the DSS.
2. The RDSS and DSS were found to have cumulative freshwater outputs of 4.9 L/m^2^/day and 2.5 L/m^2^/day, respectively. For RDSS and DSS, the highest energy efficiencies were 39.5% and 27.4%, respectively, whereas the corresponding exergy efficiencies were 3.2% and 4.7%.
3. The solar PV panel greatest power output during the tests was 47.1 W. The highest efficiency levels in the RDSS were 14.1% for electrical, 37.1% for thermal, 88.5% for heating elements, 52.1% for overall energy, and 5.9% for overall exergy.
4. The RDSS computed CPL was 0.019 $, while DSS was 0.023 $. The PBP for RDSS and DSS were 3.6 and 4.4 months, respectively which demonstrating the financial benefit and long-term viability of RDSS. A more economically feasible technology was shown by the 19.6% higher PCR of RDSS compared to DSS. While raising the interest rate from 2.5% to 12.5% for a 25-year system increased production costs by around 166.6%, extending the duration of RDSS from 5 to 25 years decreased production costs by about 73.1% at a 2.5% interest rate.
5. The CCE values for RDSS and DSS were 11.3 and 5.7 tons, respectively, whereas the anticipated CO₂ emissions were 11.3 and 5.7 tons. There was a 536.7% increase in CO₂ mitigation and a 537.4% rise in CCE when the system lifespan was extended from 5 to 25 years.
6. The RDSS sustainability index was roughly 1.6% higher than DSS which indicating better operational performance and efficiency over the long run. The water produced by both systems complied with WHO drinking water requirements, according to distillate quality evaluations.
7. Overall, the integrating a solar PV panel with thermal components in the RDSS maximizes waste heat use and minimizes shadowing losses for sustainable and scalable water to energy generation. It is beneficial for decentralized applications especially in remote or desert places.

## Scalability and sustainability: limitations and future work

The experimental performance of the RDSS demonstrates its potential for scalable and sustainable freshwater production. However, the present study is subject to certain limitations that should be acknowledged. The experimental investigation was conducted at a single location under specific climatic conditions and over a limited testing duration. Long-term outdoor operation, seasonal variations, and material aging effects were not examined. In addition, advanced control strategies, large-scale system integration, and detailed heat-loss partitioning were beyond the scope of the current work. To address these limitations and support future scalability and sustainability, the following research directions are recommended:

- Use accessible and reasonably priced commercial materials to lower system costs and promote widespread adoption.
- By combining modular solar stills with PCM technology, you may increase thermal and financial performance through proportionate scaling, reduced material and space requirements, and innovative designs.
- Using renewable technologies in absorbers and condensers, like solar concentrators or hybrid nanofluids, can optimize freshwater output, maintain constant thermal input, and improve heat transfer.
- Use IoT and AI-powered control systems to track and improve system efficiency, auxiliary heating, and solar PV panel performance in real time.
- Conduct feasibility studies in various climates with multi-day observations and locations to confirm adaptation. To promote adoption and commercialization, collaborate with politicians, industry stakeholders, and funding agencies to use incentives, subsidies, and carbon credits.

## Supplementary Information

Below is the link to the electronic supplementary material.

Supplementary Material 1

## Data Availability

The data that support the findings of this study are available from the first author on reasonable request ( [ramasamy_dhivagar@yu.ac.kr](mailto: ramasamy_dhivagar@yu.ac.kr) ).
