# Supplementary material for: Sustainable and scalable double slope solar still: a comprehensive experimental assessment of energy, exergy, economic, environmental, sensitivity and distillate performance
Source: Sci Rep. 2026 Feb 26;16:11168. doi: 10.1038/s41598-026-40989-3 (PMC13046745; doi:10.1038/s41598-026-40989-3)
Supplement: Supplementary file 1 — Supplementary Material 1 [file 41598_2026_40989_MOESM1_ESM.docx]

**Nomenclature**

***Acronyms***

DSS Double slope solar still

PCM Phase change material

PV Photovoltaic panel

RDSS Reconfigured double slope solar still

WHO World Health Organization

***Symbols***

$AC$ Total annual cost ($/year)

$A_{dss}$ Area of DSS (m^2^)

$AMC$ Annual maintenance cost ($/year)

$A_{pv}$ Area of PV panel (m^2^)

$A_{sh}$ Area of shadow (m^2^)

$ASV$ Annual salvage value ($/year)

*a* Instrument accuracy (-)

$CC$ Capital cost ($)

$CCE$ Carbon credit earned ($)

$CPL$ Cost of production ($/L)

$C_{p}$ Specific heat capacity (kJ/kg ºC)

$CRF$ Capital recovery factor (-)

$E_{\mathrm{in}}$ Embodied energy input (KWh)

$E_{\mathrm{out}}$ Embodied energy output (KWh)

$\dot{E}_{x,in}$ Exergy input rate (W)

$\dot{E}_{x,out}$ Exergy output rate (W)

$FAC$ Fixed annual cost ($/year)

$f_{dir}$ Fraction of direct beam (%)

$f_{sh}$ Fraction of shadow (%)

$h$ Height of PV panel (m)

$I_{si}$ Solar irradiation (W/m^2^)

$I_{sc}$ Short-circuit current (A)

$i$ Interest rate (%)

$L$ Latent heat of evaporation (kJ/kg)

$L_{sh}$ Length of shadow (m)

$LT$ Life time of solar still (years)

$m_{sw}$ Hourly saline water (L/h)

$m_{w}$ Hourly freshwater productivity (L/h)

$N_{{CO}_{2}}$ Net CO_2_ mitigation (tons)

$n$ Lifespan of the solar still (years)

$PBP$ Payback period (years)

$PCR$ Profit-cost ratio (-)

$P_{d}$ Annual freshwater productivity (L/year)

$P_{max}$ Maximum electrical power (W)

$POW$ Water price ($/L)

$R_{{CO}_{2}}$ International carbon cost ($/ton)

$R_{loss}$ Radiation loss (%)

$S$ Salvage value ($/year)

$SFF$ Sinking fund factor (-)

$\mathrm{SI}$ Sustainability index (-)

$T_{a}$ Ambient temperature (°C)

$T_{i}$ Inlet saline water temperature (°C)

$T_{o}$ Preheating temperature (°C)

$T_{s}$ Sun temperature (6000 K)

$T_{w}$ Water temperature (°C)

$UAB$ Cost of benefit ($/year)

$u$ Standard uncertainty (-)

$V_{oc}$ Open-circuit voltage (V)

$w$ Width of PV panel (m)

***Greek symbols***

$\eta_{dss}$ Energy efficiency of DSS (%)

$\eta_{he}$ Energy efficiency of heating element (%)

$\eta_{o-ee}$ Overall energy efficiency (%)

$\eta_{pv}$ Electrical efficiency of PV panel (%)

${}_{o-ex}$ Overall exergy efficiency (%)

$\eta_{tpv}$ Thermal efficiency of SPV panel (%)

*θ* Tilt angle of PV panel (^0^)

*α* Solar elevation angle (^0^)
